# Aqua–Chloro Complexes of Plutonyl in Hydrochloric Aqueous Solutions: Theoretical Study of Individual Contributions to XAS and UV–Vis Spectra

**DOI:** 10.1021/acs.inorgchem.6c02837

**Published:** 2026-08-09

**Authors:** Gema Raposo-Hernández, Rafael R. Pappalardo, Andrea Melchior, Enrique Sánchez Marcos

**Affiliations:** † Department of Physical Chemistry, University of Seville, 41012 Seville, Spain; ‡ Dipartimento Politecnico di Ingegneria e Architettura, Laboratorio di Tecnologie Chimiche, Università di Udine, 33100 Udine, Italy

## Abstract

Plutonium speciation in high-salinity media remains difficult to characterize, yet it largely governs the geochemical fate of PuO_2_^2+^ in marine environments. EXAFS and UV–vis measurements established that the evolution of specific spectral features correlates with the formation of successive aqua–chloro complexes. These signals represent a superposition of coexisting species in equilibrium, masking the individual contributions of each complex. In this work, we address this limitation with a combination of the nuclear ensemble approach (NEA) and high-level quantum-mechanical calculations, accounting for the relativistic Hamiltonian, spin–orbit coupling, and electron correlation, to isolate these overlapping contributions of the [PuO_2_(H_2_O)_*m*_Cl_*n*_]^2–*n*^ series and clarify the observed plutonyl speciation. The sensitivity of our methodology is quantified by comparing Wigner sampling against classical molecular dynamics (MD) trajectories. The results establish UV–vis spectroscopy as a high-resolution probe of the equatorial ligand field and provide a molecular basis for Pu­(VI) speciation in saline environments.

## Graphical abstract

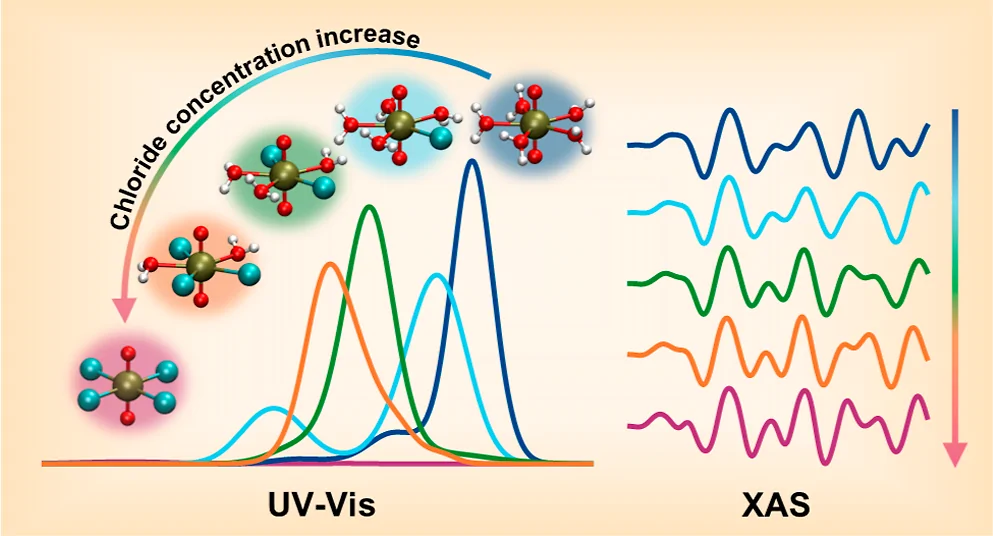

## Introduction

The ocean serves as one of the primary geochemical sinks for anthropogenic pollutants, receiving both surface runoff and atmospheric deposition.^1–4^ As global decarbonization efforts proceed, the nuclear industry represents a critical component of energy production; Europe alone operates over 100 reactors,^5^ many of them located along the coast. This geographical proximity makes seawater a potential long-term reservoir for actinide contamination, necessitating a rigorous understanding of radionuclide interactions with seawater, a chemically complex medium dominated by high ionic strength and competing inorganic ligands. This is a critical concern regarding the bioaccumulation of heavy metals and radionuclides in marine food chains.^4,6,7^

Among actinides, plutonium is of particular concern because of its persistent radiotoxicity.^8^ Its presence in marine environments comes from atmospheric tests of the 20th-century and point-source accidents, including the Chernobyl and Fukushima Daiichi disasters,^9–12^ underscoring the need to understand actinide behavior in saline media. Elevated concentrations of competing anions, such as chloride, can drastically alter radionuclide speciation, solubility, and mobility.^13^

In aqueous solution, plutonium shows redox versatility, with oxidation states from III to VII. Pu­(IV) is the predominant species in solution, but oxidizing or radiolytic environments stabilize higher oxidation states, making Pu­(VI) relevant across both the nuclear fuel cycle and environmental systems.^14,15^ In the PUREX process, plutonium is maintained in its actinyl form to facilitate its separation from minor actinides.^16^ Parallel to this industrial relevance, the plutonyl cation, PuO_2_^2+^, exhibits a strong affinity for halide ligands in saline media, as does its uranyl UO_2_^2+^ homologue.^17–22^ However, chemical intuition derived from uranyl to plutonyl is limited. The electronic structure of Pu­(VI), with two 5f electrons, is considerably more complex than that of U­(VI). The 5f^2^ configuration generates a dense manifold of low-lying excited states with strong spin–orbit coupling and pronounced multireference character. A molecular-level description of the [PuO_2_(H_2_O)_*m*_Cl_*n*_]^2–*n*^ complexation connects microscopic electronic properties with the chemical behavior of high-valence plutonium in saline environments, even though Pu­(VI) is a minor fraction of the total speciation in seawater.^23^ Previous investigations of actinyl aqua and chloro complexes have combined spectroscopic measurements with theoretical modeling,^24–30^ providing valuable structural and electronic insight while also revealing intrinsic limitations in species discrimination under equilibrium conditions.

X-ray absorption spectroscopies, in particular XANES and EXAFS, have been extensively applied to actinide systems to determine oxidation states, average coordination numbers, and metal–ligand bond distances in solution.^27,28,31^ Complementary vibrational and electronic spectroscopies, such as Raman, UV–vis, or vis–NIR, provide additional sensitivity to molecular symmetry, ligand-field effects, and electronic transitions within the 5f manifold.^32,33^ In principle, these techniques offer distinct spectral fingerprints for different coordination environments and ligand sets. However, in practice, the observed spectra frequently reflect a superposition of multiple species coexisting in equilibrium, particularly in high ligand concentration media, as is the case for concentrated chloride solutions.

As a result, although spectroscopy has firmly established the presence of chloro complexation in plutonyl systems, the precise nature and coordination of individual species remain subject to interpretation. A representative example is provided by the work of Runde et al.,^6^ who investigated the plutonyl chloride system by monitoring the evolution of UV–vis and EXAFS spectra as a function of increasing chloride concentration. The formation of successive complexes is evidenced by the gradual spectral changes observed with increasing Cl^–^ concentration. At high chloride molarities, they observed a low-intensity absorption band, which they assigned to the tetrachloro complex, [PuO_2_Cl_4_]^2–^. This assignment was further supported by Raman spectroscopy, where a vibrational mode (B_2g_) appeared at frequencies consistent with the D_4h_ symmetry found in analogous actinyl–chloro complexes. Based on these observations, a decrease in the equatorial coordination number from five to four was proposed for the tetrachloro species. Nevertheless, the coordination environment of intermediate aqua–chloro complexes could not be unambiguously resolved. In particular, the intrinsic resolution limits of EXAFS when determining coordination numbers, typically associated with uncertainties of approximately ±1, preclude definitive structural assignments, leaving a gap that requires theoretical intervention to disentangle overlapping spectroscopic signatures.

Early DFT studies reproduced structural evolution and hydration of actinyl ions,^30,34,35^ while more recent works show the importance of accounting for relativistic effects and electronic correlation in plutonium complexes.^36,37^ In Pu­(VI), the high density of excited states and significant spin–orbit coupling necessitate high-level multiconfigurational methods for chemical accuracy.

Despite the maturity of these computational methods, the theoretical reproduction of PuO_2_^2+^ absorption spectra remains a formidable challenge due to the fundamental selection rules governing f–f transitions. As established by Matsika et al.,^38^ since all low-lying electronic states for the isolated 5f^2^ actinyl ions possess the same parity (gerade), electric–dipole transitions are strictly Laporte-forbidden. The emergence of experimental intensity therefore relies on the breaking of inversion symmetry, either through the static effect of the equatorial ligand field or via vibronic coupling. Matsika et al.^38^ demonstrated that the presence of chloride ligands in the equatorial plane facilitates the mixing of states with different parity, yet their models remained limited to specific coordination geometries. More recently, Danilo et al.^39^ provided a rigorous description of the [PuO_2_(H_2_O)_5_]^2+^ electronic structure using CASPT2 and MRCI + DC methods, incorporating both the first hydration sphere and bulk solvent effects. However, a significant limitation persists in the literature: most theoretical studies rely on static optimized structures. In such high-symmetry configurations (e.g., D_5h_ or D_4h_), the parity-breaking mechanisms are insufficiently captured, leading to calculated oscillator strengths that are either vanishingly small or identically zero. As a result, while transition energies can be predicted with high precision, direct simulation of experimental spectral bands has not been fully realized. This challenge is further highlighted by the recent findings of Neill et al.,^21^ who demonstrated through a combined experimental and theoretical approach that equatorial halide ligands induce a significant electronic reorganization via an energy-match driven mechanism and a “pushing from below” effect on the frontier orbitals. Such perturbations suggest that the formation of successive plutonyl–chloro complexes should lead to substantial modifications in the electronic structure, potentially affecting both the transition energies and the intensities of the spectral signals. However, as these effects are inherently coupled to the local symmetry of the complex, their accurate reproduction in simulated spectra requires a methodology that moves beyond the static equilibrium approximation.

Since f–f transitions are formally Laporte-forbidden in centrosymmetric geometries, their nonzero intensity in solution arises from the symmetry-breaking distortions of the coordination environment. Static calculations from a single optimized structure cannot capture this effect, so we employ a nuclear ensemble approach based on Wigner sampling, which has proven effective for the absorption spectra of heavy-metal aqua ions and complex ligand environments.^40–44^ High-level quantum mechanical (QM) calculations are performed over a representative distribution of geometries, generating ensemble-averaged spectra for the [PuO_2_(H_2_O)_*m*_Cl_*n*_]^2–*n*^ series. This enables a direct simulation of the absorption bands for each individual complex, separating their contributions to disentangle the complex speciation of plutonium in chloride-rich media.

## Methodology

### QM Calculations

Optimizations and frequency calculations were needed for Wigner sampling at room temperature (300 K). They were done by means of the second-order Møller–Plesset Perturbation Theory (MP2). The basis sets used are SD­(60,MWB)/DEF-TZVP^45–47^ for Pu and aug-cc-pVTZ^48^ for Cl, O, and H.^48,49^ Solvation effects beyond the first coordination shell were accounted for using the CPCM solvation model.^50^

To construct the electronic spectrum, we adopted a strategy similar to that employed in previous works, where aqua complexes of Ce^3+^ and U^4+^ were investigated.^40,41^ This involved the systematic computation of the electronic structure for the plutonyl ion in its pentaaqua form, [PuO_2_(H_2_O)_5_]^2+^, and its subsequent series of aqua–chloro complexes, [PuO_2_(H_2_O)_*m*_Cl_*n*_]^2–*n*^ (where (*m*, *n*) = (4,1), (3,2), (2,3), and (0,4)).

The ground- and excited-state wave functions were derived from state-average Complete Active Space Self-Consistent Field (CASSCF)^51–53^ calculations, including two electrons distributed over the seven 5f orbitals, resulting in 21 triplet states and 28 singlet states. Dynamical correlation was incorporated using the N-electron valence state perturbation theory NEVPT2,^54–56^ keeping the 1s oxygen atomic orbitals and all plutonium core orbitals below the 5d orbitals frozen. In addition, CASPT2 ^57–59^ calculations were performed using the ionization potential electron affinity (IPEA)-corrected zeroth-order Hamiltonian^60^ while keeping the same frozen core definition as described above. An imaginary shift of 0.25*E*_h_ was used to avoid intruder states.

The relativistic Hamiltonian employed is the Exact Two-Component Hamiltonian (X2C), which provides an accurate description of the relativistic effects on actinoids.^61–65^ This method utilizes the X2C Hamiltonian with the X2C-TZVPall basis sets^66,67^ for O and H and the cc-pVQZ-X2C basis set^68^ for Pu. Several authors^69,70^ have performed calculations of properties depending on relativistic effects and spin–orbit couplings on actinides. They used specific basis sets of triple-ζ quality (cc-pVTZ-X2C^69^ and ANO basis sets truncated to the triple-ζ level^70^). They have shown good results when compared to the experimental data. The inclusion of spin–orbit (SO) coupling was done using mean-field approximation (SOMF)^71^ combined with the resolution of the identity (RI-SOMF­(1X)). An example of an input file is included in the Supporting Information (SI). All calculations were conducted using the ORCA 6.1 software.^72,73^

### Statistical Sampling: Molecular Dynamics and Wigner Distribution

In the context of PuO_2_^2+^ UV–vis spectroscopy, the relevant excited states correspond to Laporte-forbidden f–f transitions. It is essential to sample distorted molecular configurations to capture the intensity that arises from symmetry breaking and ligand-field splitting. Two complementary sampling methods were used to generate or to extract statistically significant sets of geometry for spectrum generation: Wigner sampling and classical molecular dynamics (MD) simulations. Classical MD simulations are a valuable technique for statistical sampling, especially when the force field (FF) parameters are derived from high-level quantum mechanical (QM) calculations. However, this approach presents some limitations, including the elaborated FF parametrization and the requirement for system-specific parameter sets, which restricts its transferability to the successive chloro species. Our group developed a set of FFs for describing actinyl AnO_2_^2+^ cations, with An = U­(VI), Np­(V,VI), Pu­(VI), and Am­(VI), in water.^74–76^ A series of snapshots were extracted from a 5 ns simulation of a cubic box containing the actinoid and 1495 TIP4P water molecules. The time step of 1 fs was selected, and the average temperature and pressure were kept constant at 300 K and 1 atm using the Nosè–Hoover barostat. Additional methodological details of the MD simulation can be found in refs 74–76 .

Another widely used technique for generating distorted molecular geometries is Wigner sampling,^77–79^ which creates an ensemble of structures based on the harmonic normal vibrational modes obtained at the potential energy surface (PES) minimum of the target system.^80^ This method circumvents the need for classical FFs or computationally demanding ab initio molecular dynamics; it relies on the harmonic description, which can be less accurate for systems with low-frequency modes or high conformational flexibility. Wigner sampling is well-suited for studying the complexation and speciation of heavy metals in aqueous environments, where metal–ligand interactions are predominantly ionic and the degree of anharmonicity is minimal. In this work, Wigner distributions were generated at 300 K based on the optimized structures and harmonic vibrational frequencies, using the *wigner.py* utility from the SHARC package.^81^ The frequencies below 250 cm^–1^ for the aqua ion and 200 cm^–1^ for the aqua chloro species were discarded to avoid artificial O–H displacements originating from the description of torsional modes.^82,83^

### Methodological Approach to Calculating Average EXAFS, XANES, and UV–Vis

The average EXAFS spectra were computed from 500 individual spectra obtained from sampled configurations. Pu L_III_-edge k^3^-weighted spectra were computed including multiple scattering (MS) up to four legs and a maximum path length of 6 Å. Details regarding the generation of EXAFS using self-consistent calculation of the wave function, with the Hedin–Lundqvist exchange correlation functional, can be found in prior works.^84–86^ An example of the FEFF input file to compute the EXAFS signal is provided in the SI.

Since the absolute intensity scale of the experimental EXAFS data was not explicitly provided in the source literature,^21^ the experimental signal was scaled to match the amplitude of the theoretical spectrum calculated with an amplitude reduction factor (S_0_^2^) of 1.0. The energy correction of the threshold, Δ*E*_0_, is fixed to −2.0 eV to make the first oscillation of the theoretical spectra match the first oscillation of the aqua ion experimental spectrum. For the calculations of XANES spectra, the number of individual spectra averaged was reduced to 100 sampled configurations, as this part of the XAS spectrum is less sensitive to geometrical fluctuations. In the SCF computations, the Hedin–Lundqvist exchange correlation functional provided an ideal balance between the intensity of the white line and subsequent resonances. No shift in energy was applied to the theoretical spectra to match the experimental edge position. An input file for the XANES computation is also given in the SI. EXAFS and XANES spectra have been simulated using the structural information and the theoretical scattering phases and amplitude functions computed by the ab initio FEFF code (v.10.0.0).^87,88^

The average UV–vis spectra were reconstructed from 100 individual spectra using the Gaussian Mixture Model-Nuclear Ensemble Approach (GMM-NEA).^89^

## Results

### Structural Characterization

To correlate the structural evolution of the plutonyl core with its spectral response in saline environments, we first characterized the variations in the equatorial coordination sphere induced by chloride complexation. Tables 1 and 2 benchmark the MP2-optimized geometries of the [PuO_2_(H_2_O)_5_]^2+^ aqua ion and its chloro derivatives, [PuO_2_(H_2_O)_*m*_Cl_*n*_]^2–*n*^, against the available experimental and theoretical literature, reflecting the spread in reported coordination environments. A representation of the optimized structures is included in Figure S1 of the SI.

**Table 1:** Summary of Pu–O_yl_ and Pu–OH_2_ Average Distances and Coordination Numbers (CNs) Obtained from Experiments and Geometry Optimizations for [PuO_2_(H_2_O)*n*]^2+^ Aqua Ions

method (solvation)	*R*_Pu–Oyl_ (Å)	*R*_Pu–OH_2__(Å)	CN	ref
MP2 (CPCM)	1.728	2.397	5	this work
MD (Explicit)	1.71	2.45	5	^26^
B3LYP	1.71	2.46	5	^26^
NEVPT2	1.76	2.43	5	^26^
B3PW91 (CPCM)	1.70	2.42	5	^90^
BP86 (Microsolvated)	1.76	2.40–2.41	5	^34^
PBE(PCM)	1.748	2.441	5	^30^
B3LYP (PCM)	1.718	2.435	5	^30^
EXAFS	1.74	2.42	4.4	^91^
EXAFS	1.74	2.45	-	^6^
EXAFS	1.76	2.41	5.3	^21^
XANES	1.75	2.41	4.5	^31^

**Table 2:** Summary of Pu–O_yl_, Pu–OH_2_, and Pu–Cl Average Distances Obtained from Experiments and Geometry Optimizations for [PuO_2_(H_2_O)_*m*_Cl_*n*_]^2–*n*^ Aqua–Chloro Complexes

method (solvation)	*R*_Pu–Oyl_ (Å)	*R*_Pu–OH_2__(Å)	CN_OH2_	*R*_Pu–Cl_ (Å)	CN_Cl_	refs
MP2 (CPCM)	1.732	2.421	4	2.644	1	this work
MP2 (CPCM)	1.735	2.451	3	2.662	2 (trans)	this work
MP2 (CPCM)	1.735	2.456	3	2.658	2 (cis)	this work
MP2 (CPCM)	1.737	2.477	2	2.688	3 (trans)	this work
MP2 (CPCM)	1.737	2.494	2	2.691	3 (cis)	this work
MP2 (CPCM)	1.739	2.502	1	2.728	4	this work
MP2 (CPCM)	1.741	-	0	2.645	4	this work
B3PW91 (CPCM)	1.77	2.59	4	2.98	1	^90^
B3PW91 (CPCM)	1.72	2.50	3	2.66	2	^90^
BP86 (Microsolvated)	1.79	-	0	2.67–2.68	4	^34^
EXAFS ([Cl^–^] 2M)	1.75	2.43	4.5	2.69	0.3	^21^
XANES ([Cl^–^] 4M)	1.75	2.43	2.7	2.75	1.3	^31^
EXAFS ([Cl^–^] 5M)	1.75	2.43	-	2.75	-	^6^
EXAFS ([Cl^–^] 6M)	1.75	2.42	3.6	2.73	1.3	^21^
XANES ([Cl^–^] 16M)	1.75	2.49	2.6	2.70	2.4	^31^
EXAFS ([Cl^–^] 16M)	1.75	2.49	-	2.70	-	^6^

For the pentaaqua species, the calculated axial Pu–O_yl_ distance (1.728Å) is slightly shorter than the experimental range of 1.74 Å to 1.76 Å determined by EXAFS and XANES.^6,21,91^ This underestimation is typical of the MP2 performance in actinyl systems, arising from the treatment of subvalence correlation. Nevertheless, the equatorial Pu–OH_2_ bond length of 2.397Å aligns with the lower bound of experimental values of 2.41 Å to 2.45 Å, providing a consistent structural baseline for the subsequent substitution series.

Substitution of equatorial water by chloride ligands expands the coordination shell. The Pu–OH_2_ distance increases to 2.502 Å in the tetrachloride complex, while Pu–Cl distances evolve from 2.644 Å to 2.728 Å. These trends correlate with the results reported by Neill et al.,^21^ Conradson et al.,^31^ and Runde et al.,^6^ where Pu–Cl distances within the 2.69 Å to 2.75 Å range were observed at high salinity. As the number of chloride ligands (n) increases in the [PuO_2_(H_2_O)_*m*_Cl_*n*_]^2–*n*^ series, we observe a subtle but consistent elongation of the Pu–O_yl_ bond, which reaches 1.741 Å in the tetrachloro complex. This trend comes from the stronger equatorial donation from the anionic chloride ligands compared to the neutral water molecules; this higher electron density on the Pu center slightly weakens the axial Pu–O_yl_ interaction. The equatorial Pu–Cl bond lengths exhibit an expansion as the coordination sphere becomes more crowded. In the intermediate species, where the total coordination number (CN) remains 5, the Pu–Cl distances range from 2.644 Å to 2.728 Å. This expansion is driven by both the larger ionic radius of Cl^–^ and the increasing steric repulsion between ligands in the equatorial plane.

The most significant structural transition occurs with the formation of the tetrachloro species, [PuO_2_Cl_4_]^2–^. While the hydrated chloro complexes favor a pentacoordinated equatorial plane, the anhydrous tetrachloro complex has a CN of 4, with a D_4h_ symmetry. The decrease in coordination number with anionic ligands in the first coordination shell has been reported in the evolution of the CN of Po^4+^ hydrates when water molecules are replaced by hydroxyl anions.^92,93^ The change in coordination number is accompanied by a notable contraction of the Pu–Cl bonds to 2.645Å as the loss of the fifth equatorial ligand (water) relieves the steric repulsion. The structural reorganization from 5 to 4 ligands provides a molecular basis for the Raman B_2g_ vibrational mode identified by Runde et al.^6^ at high chloride concentrations. This transition from D_5h_ to D_4h_ not only dictates the vibrational modes of the complex but also changes the symmetry-allowed transitions governing the electronic absorption spectra, as will be discussed in the following sections.

### EXAFS

To validate the MP2 Wigner sampled ensemble, the theoretical k^3^-weighted EXAFS spectrum was compared with experimental data^21^ for the [PuO_2_(H_2_O)_5_]^2+^ aqua ion (Figure S2). The computed signal reproduces the experimental phase and amplitude, particularly within the characteristic 6 Å^–1^ to 9 Å^–1^ *k* region. The good agreement in the oscillation frequency confirms that both the Pu–O_yl_ and Pu–OH_2_ bond lengths are accurately captured by the sampling approach, providing a strong foundation for the subsequent analysis of the chloro-substituted series.

The evolution of the experimental EXAFS spectra^21^ as a function of chloride concentration (Figure 1) is characterized by the changes in the 7 Å^–1^ to 9 Å^–1^ region. In the aqua ion case, [PuO_2_(H_2_O)_5_]^2+^, the spectral profile in this range is governed by the interference between the plutonyl oxygen atoms Pu–O_yl_ and the water ligands Pu–OH_2_ paths, as shown in Figure S3 of the SI. As chloride ligands substitute water molecules, the expansion of the equatorial shell, shown in Table 2, modulates the phase and position of the single-scattering (SS) signals, directly affecting the k ∼7 Å^–1^ shoulder, as shown in the overall spectral trends observed in Figure 2.

**Figure 1:**
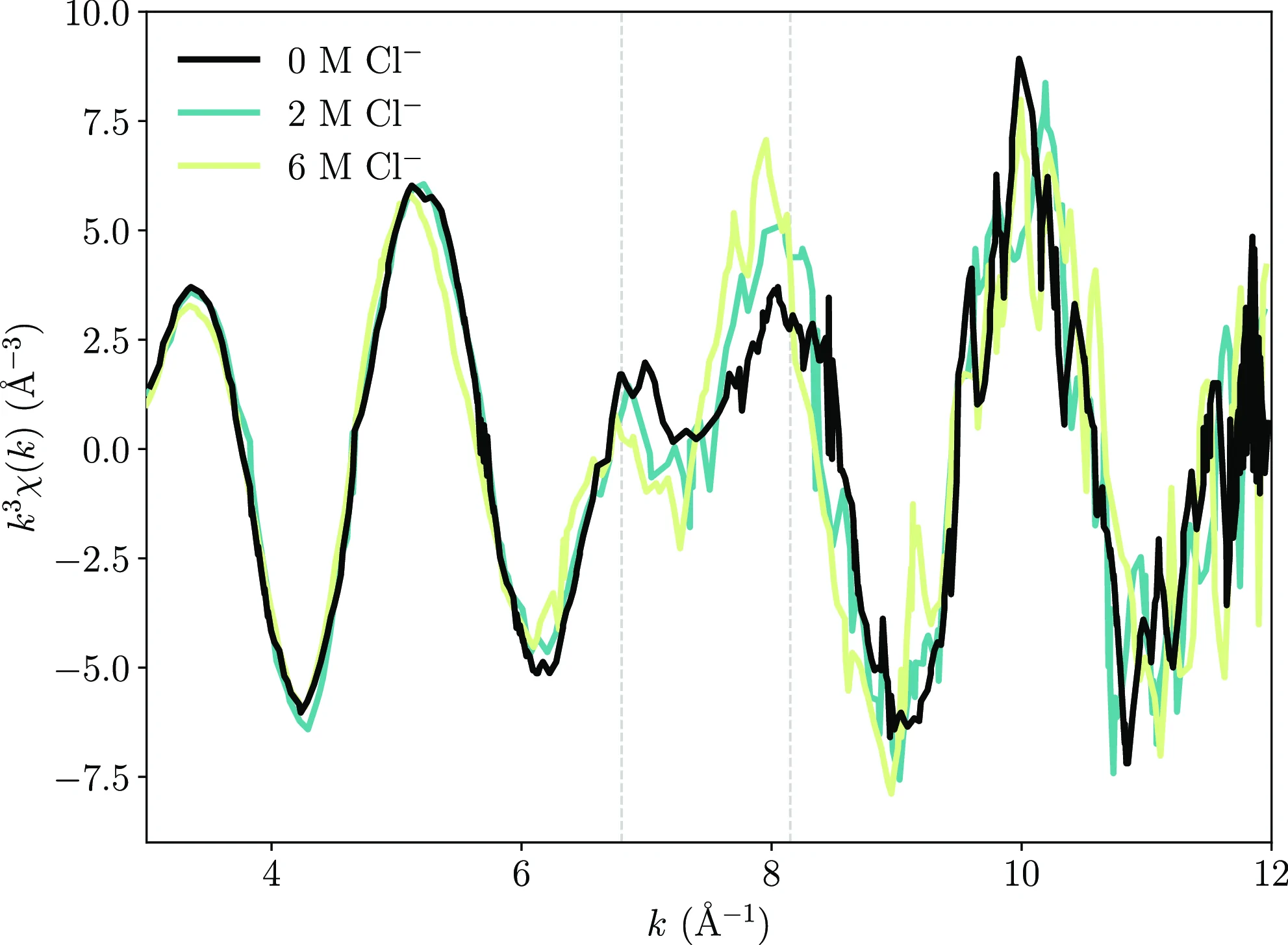
Evolution of experimental EXAFS spectra as a function of chloride concentration adapted from Neill et al.,^21^ Copyright 2025 American Chemical Society, ([PuO_2_^2+^] = 5 mM). Vertical lines at 6.8 Å^–1^ and 8.15 Å^–1^ are included as visual references.

**Figure 2:**
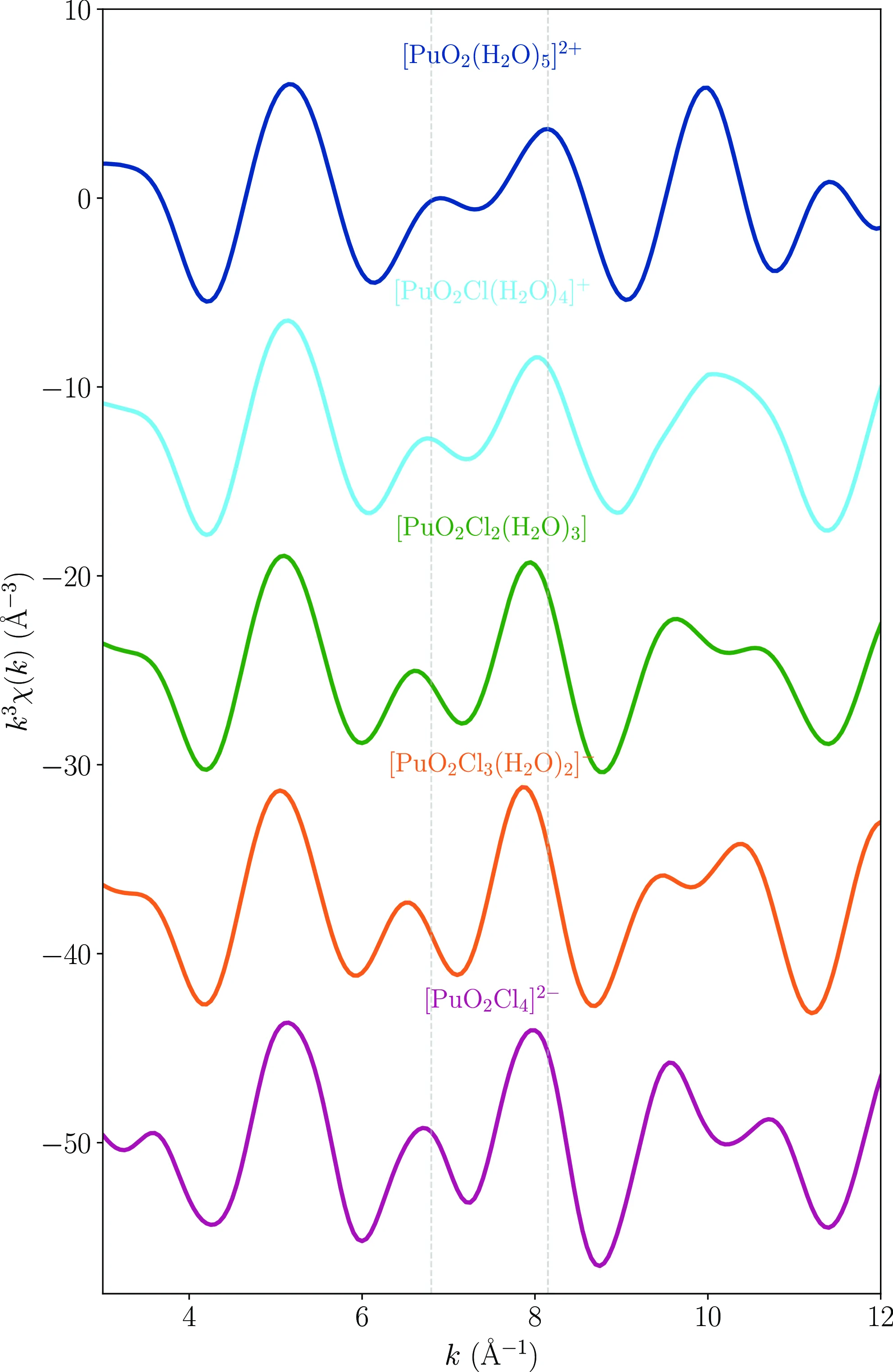
Theoretical EXAFS spectra of the [PuO_2_(H_2_O)_*m*_Cl_*n*_]^2–*n*^ series. Vertical lines at 6.8 Å^–1^ and 8.15 Å^–1^ are included as visual references. The $\left[{PuO}_{2}{\left(H_{2}O\right)}_{3}{Cl}_{2}\right]$ and ${\left[{PuO}_{2}{\left(H_{2}O\right)}_{2}{Cl}_{3}\right]}^{-}$ spectra correspond to the *trans* isomer; the results with the *cis* isomer are equivalent.

Analysis of the individual SS contributions for $\left[{PuO}_{2}{\left(H_{2}O\right)}_{3}{Cl}_{2}\right]$ , plotted in Figure 3, reveals that the Pu–Cl path exhibits a scattering frequency similar to that of the Pu–OH_2_ path. In the *k* ∼ 8Å^–1^ region, all three primary contributors (Pu–O_yl_, Pu–OH_2_, and Pu–Cl) undergo constructive interference, as evidenced by the alignment of their oscillatory maxima. The substitution of water by chloride introduces a heavier scatterer that increases the amplitude of the total oscillation, affecting the oscillation intensity at k ∼ 8 Å^–1^. At the same time, this substitution induces an evolution of the coordination shell distances. The Pu–Cl bonds, longer than their Pu–OH_2_ counterparts, reach a maximum elongation in the trichloro complex before contracting in the tetrachloro species as the total coordination number (CN) shifts from 5 to 4. As shown across the full series (Figure 4), these combined effects produce a progressive enhancement of the feature at k ∼ 8 Å^–1^ and the restructuring of the k ∼ 7 Å^–1^ feature, which are the structural fingerprint of the equatorial reorganization induced by chloride coordination.

**Figure 3:**
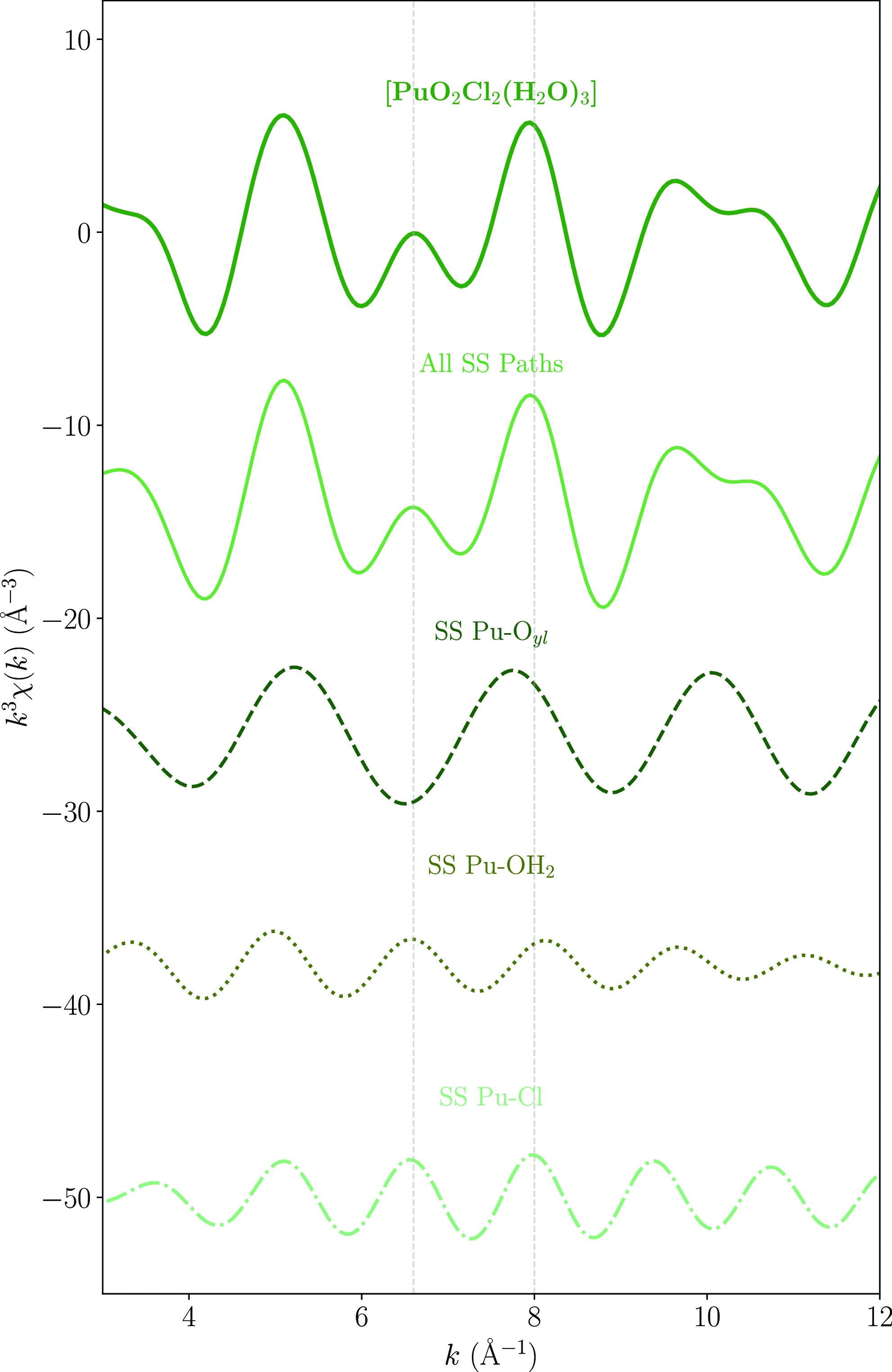
Theoretical EXAFS spectrum of $\left[{PuO}_{2}{\left(H_{2}O\right)}_{3}{Cl}_{2}\right]$ and the contribution of single scattering paths. To aid in correlating spectral features with their specific single scattering (SS) origins, vertical markers are included at 6.6 Å^–1^ and 8 Å^–1^ as a visual reference.

**Figure 4:**
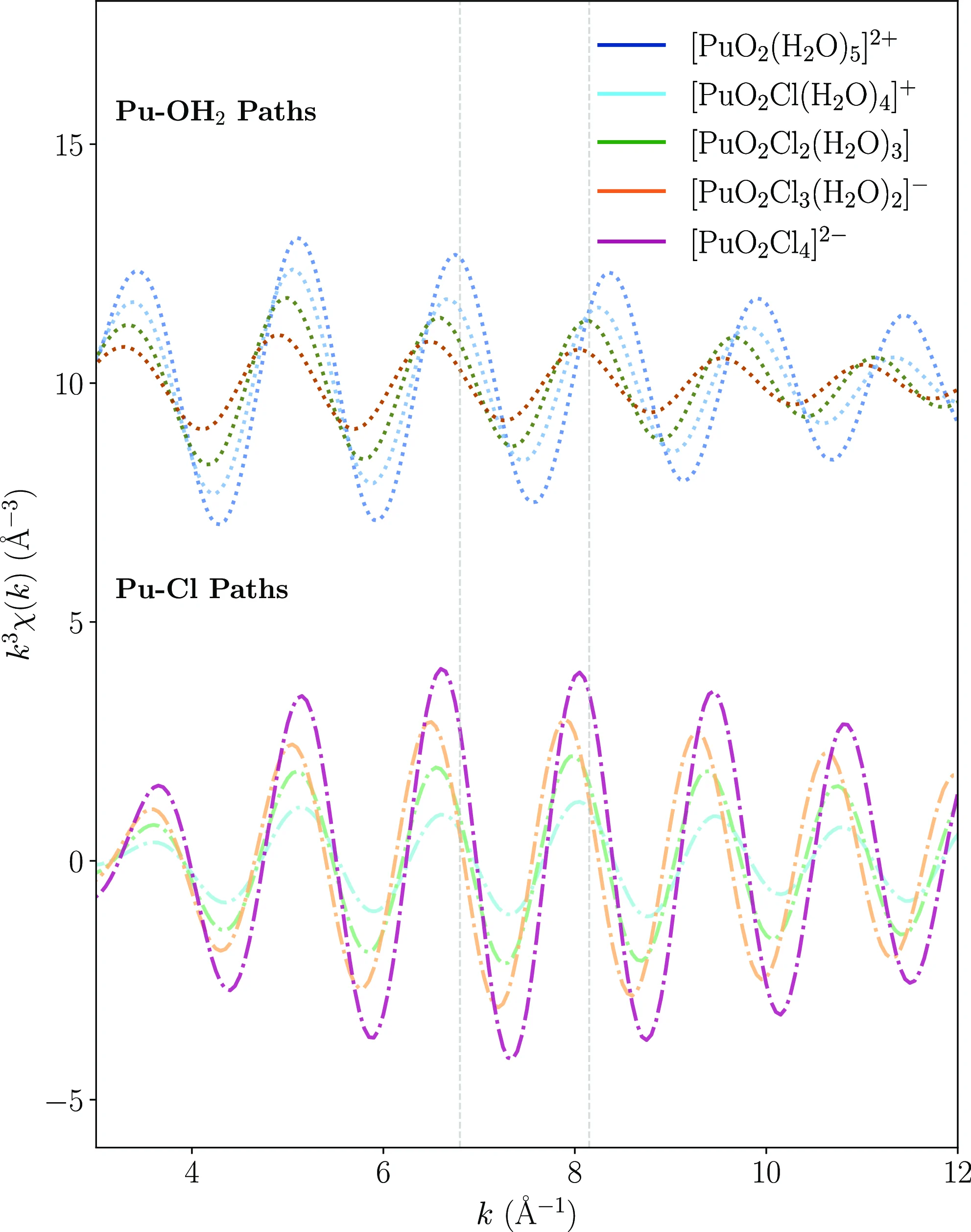
Theoretical EXAFS contributions of Pu–OH_2_ and Pu–Cl single scattering paths. Consistent with previous figures, the vertical lines at 6.8 Å^–1^ and 8.15 Å^–1^ are visual references.

### XANES

To evaluate the Pu­(VI) speciation in chloride media, we analyzed the evolution of the XANES spectra as a complementary diagnostic tool. As shown in Figure 5, the experimental L_III_-edge spectra are somewhat insensitive to increasing chloride concentration. The position of the absorption edge and the white-line intensity show only minor fluctuations, indicating that the Pu­(VI) oxidation state and the *trans*-dioxo core symmetry are strictly preserved throughout the substitution series.

**Figure 5:**
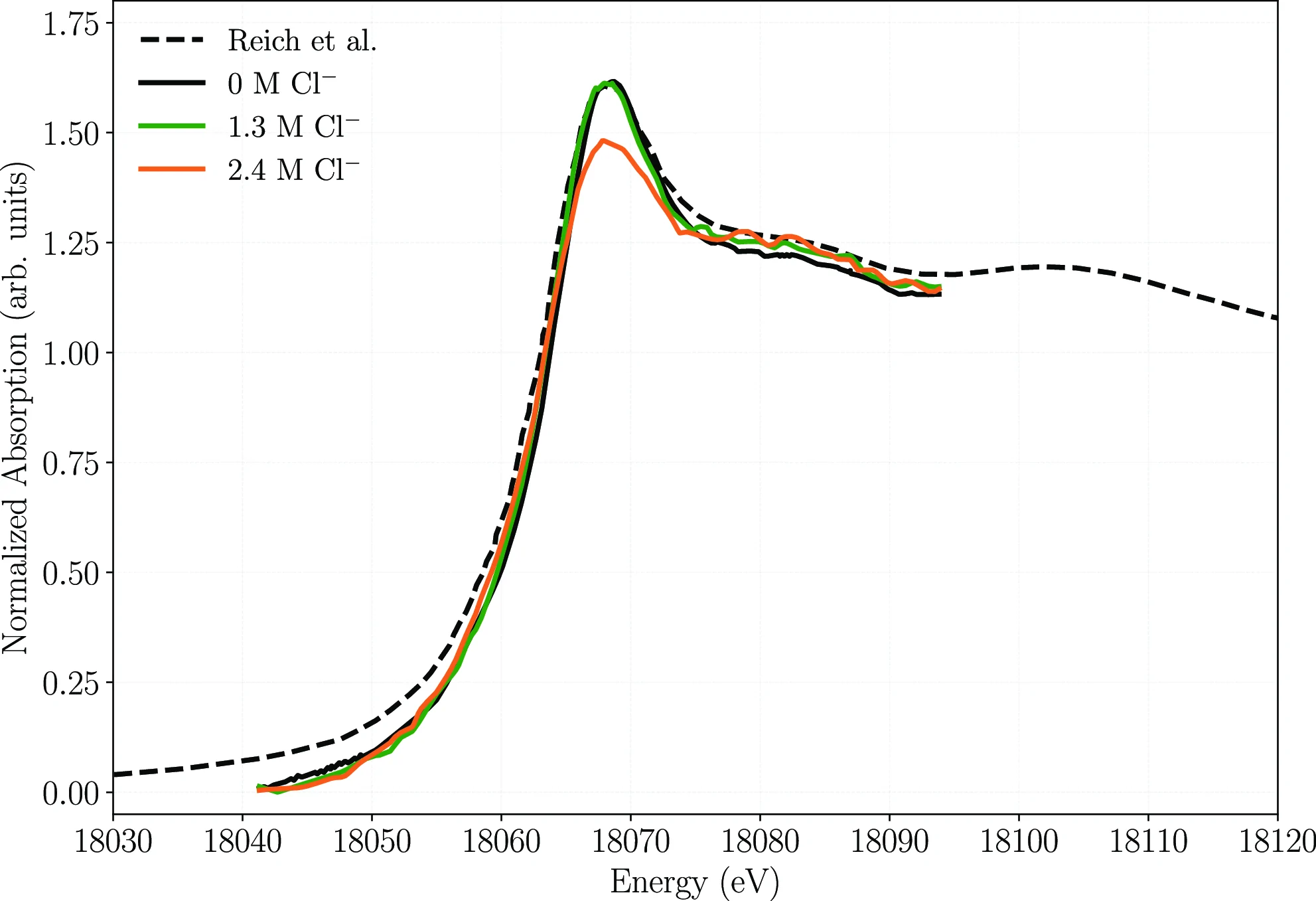
Evolution of experimental XANES spectra as a function of chloride concentration extracted from ref 31, Copyright 2004 American Chemical Society. The XANES spectrum of the aqua ion [PuO_2_(H_2_O)_5_]^2+^ from Reich et al.^91^ is also included.

However, a closer examination of the experimental series reveals some sensitivity to the chloride concentration. Notably, the spectral variation observed when increasing the salinity from 1.3 to 2.4 M Cl^–^ (green vs orange lines) is more pronounced than the change recorded between the aqua ion (0 M) (black vs green lines) and the 1.3 M condition. This observation suggests that the transition toward higher-order chloro complexes (such as the di- or trichloro species) induces a more significant perturbation of the plutonium electronic density than the initial monosubstitution.

The theoretical XANES spectra, computed for the [PuO_2_(H_2_O)_*m*_Cl_*n*_]^2–*n*^ series (Figure 6), follow the same trend observed in the experimental spectra and reproduce the shoulder on the high-energy side of the white line (18,085 eV), which is characteristic of the multiple scattering (MS) processes within the Pu–O_yl_ bonds.^94^

**Figure 6:**
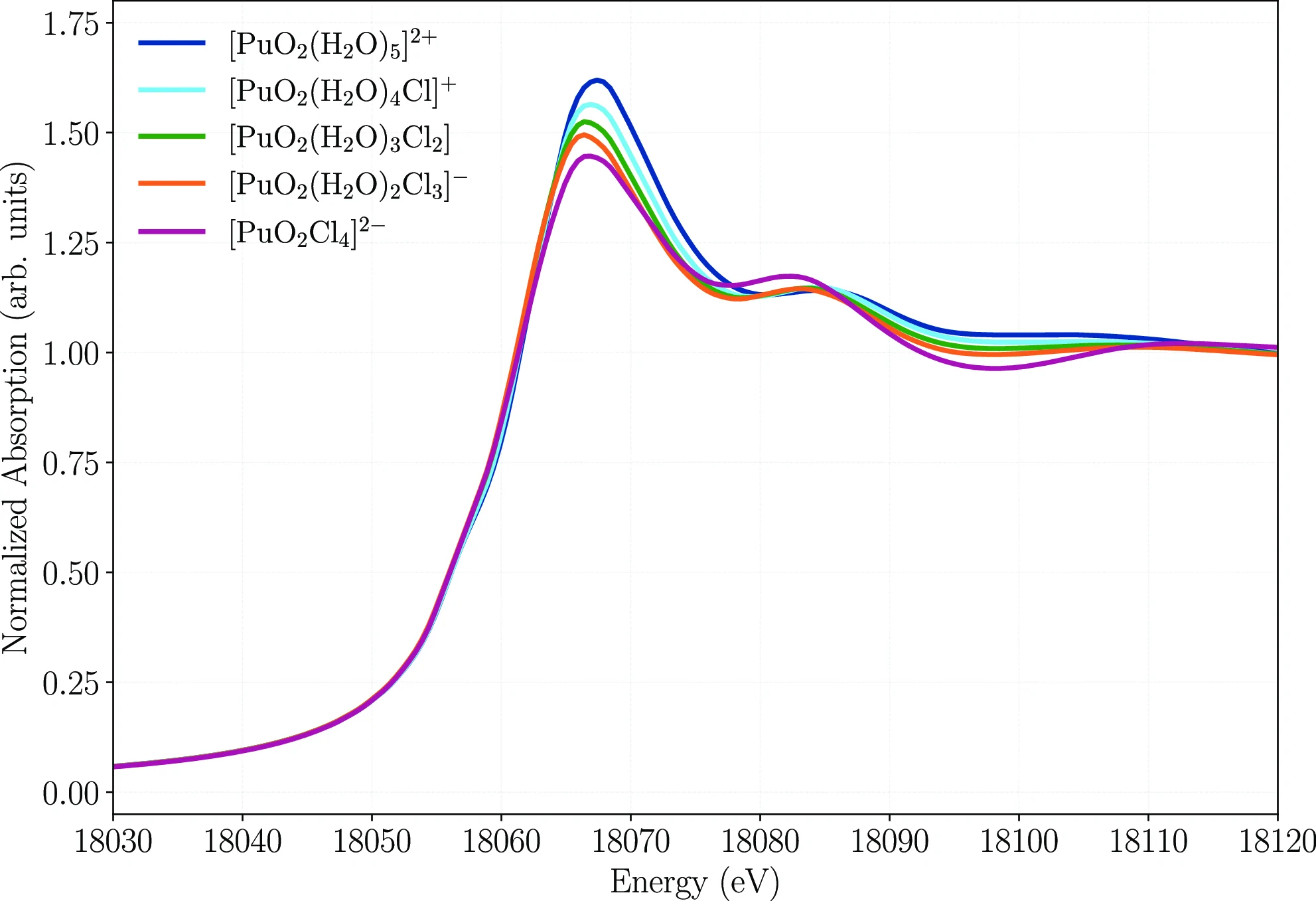
Theoretical XANES spectra of the [PuO_2_(H_2_O)_*m*_Cl_*n*_]^2–*n*^ series.

Neill et al.^21^ have recently recorded the Pu M4-edge of the plutonyl aqueous complex at 0 and 6 M of hydrochloric acid. Figures S4 and S5 in SI show a comparison of the experimental spectra with the simulated ones for the plutonyl pentahydrate and one of the aqua–chloro complexes. The chloride concentration increase is qualitatively reproduced in both the edge position and the shift of the second maximum at about 3977 eV.

### UV–Vis

For the characterization of the 5f^2^ electronic manifold in the plutonyl ion, we employed a CASSCF­(2,7) reference space comprising the two valence electrons distributed among the seven 5f orbitals. Two perturbative schemes were considered: NEVPT2 and CASPT2. To assess the consistency of our approximation, we compare the results to the experimental transitions reported by Edelstein^95^ for the case of the plutonyl aqua ion. To prevent the artificial splitting of degenerate states, vertical transitions were computed by using a D_5h_-symmetrized structure for [PuO_2_(H_2_O)_5_]^2+^, allowing for a consistent assignment based on the irreducible representations of the point group.

The comparison in Table 3 shows that NEVPT2 and CASPT2 agree closely in the near-infrared (NIR) region, with a nonlinear divergence that emerges as the manifold progresses toward higher energies, with the NEVPT2 values being consistently higher than their CASPT2 counterparts. Both methods show a systematic blue shift relative to the experimental data. This overestimation is well-documented in the multireference treatment of actinyl systems^39^ and reflects the inherent difficulty of second-order perturbation theory to fully recover the dynamic correlation between the 5f electrons and the 6*s*/6p subvalence shells. The 5d, 6s, and 6p subvalence shells were kept frozen during the perturbative step. Unfreezing these shells could partially recover core–valence correlation, but the standard cc-pVQZ-X2C basis set^68^ is primarily optimized for valence correlation. Incorporating subvalence correlation without the corresponding core-consistent (CV) functions would not yield an improved description while significantly increasing the computational cost.

**Table 3:** Comparison of Experimental and Calculated Electronic Transitions (cm^–1^) for the [PuO_2_(H_2_O)_5_]^2+^ Aqua Ion^d^

Edelstein et al.^a^		Edelstein et al.^a^		Danilo et al.^b^		This Work^c^			
State (*D*_*∞h*_)	Band	Exp	Calc	State (Ω)	Calc. (PCM)	NEVPT2	CASPT2	Scaled NEVPT2 (σ = 0.873)	Scaled CASPT2 (σ = 0.893)
*E*_4*g*_		0	0.8	4_*g*_	0	0	0	0	0
*A*_1*g*_	–	–	2143	0_*g*_^+^	2657	1828	2155	1596	1924
*E*_1*g*_	–	–	3823	1_*g*_	4914–4930	4371	4474	3816	3995
*E*_5*g*_	A	7385	7277	5_*g*_	7751–7821	7645	7649	6674	6831
*E*_1*g*_	B	10187	10228	0_*g*_^‑^	11643	11942	11538	10425	10304
*A*_1*g*_	C	10442–10526	10427	1_*g*_	11528–11543	11007	11024	9609	9844
*A*_2*g*_	D	10706	10593	0_*g*_^+^	12392	12265	11947	10708	10668
*E*_2*g*_	**F**	**12045**	**12129**	**2**_***g***_	**13656**	**13805**	**13494**	**12052**	**12050**
*E*_6*g*_	G	12643	12943	6_*g*_	14222–14228	13951	13867	12179	12383
-	H	12866	–	–	–	–	–	–	–
*E*_4*g*_	–	–	13863	–	–	–	–	–	–
*A*_1*g*_	–	–	15684	–	–	15481	15506	13515	13847
–	I	15458	–	0_*g*_^+^	18442	17941	17990	15663	16065
*E*_3*g*_	J	16055	16128	4_*g*_	18643–18685	–	–	–	–
–	K	16880	–	–	–	–	–	–	–
*E*_2*g*_	L	17799	17836	1_*g*_	20810–20834	21097	20696	18418	18483
*E*_1*g*_	–	–	18260	–	–	–	–	–	–
*A*_1*g*_	N	19111	19098	–	–	22196	21797	19377	19464
*E*_5*g*_	O	19811	19827	0_*g*_^+^	22101	24325	24533	21235	21908
*E*_2*g*_	–	–	20137	–	–	26403	24610	23049	21977
*E*_1*g*_	P	21239	21223	5_*g*_	24892–24918	26871	25215	23458	22517
*E*_4*g*_	Q	21828	21736	1_*g*_	26387–26389	27496	26125	24004	23330
*E*_1*g*_	–	–	22556	6_*g*_	29202–29300	29249	27980	25534	24986
–	–	–	–	0_*g*_^+^	36612	30191	29717	26356	26537
**Mean Absolute Error**						**2453**	**2269**	**429**	**433**

a Crystal field analysis and experimental assignment from Edelstein.^95^

b CASPT2/SO-RASSI results with implicit solvation from Danilo et al.^39^

c This work. Calculated VEE using a CASSCF­(2,7) reference. Scaled values are obtained by multiplying the raw energies by σ = 0.873 (NEVPT2) and σ = 0.893 (CASPT2) to match the energy of Band F.

d Theoretical values from this work are presented both as raw VEEs and after applying a uniform scaling factor (σ).

We applied uniform scaling factors (σ_NEVPT2_ = 0.873; σ_CASPT2_ = 0.893) to align our theoretical results with the position of the experimental Band F (12,045 cm^–1^). This transition was chosen as the reference point due to its high intensity and its sensitivity to perturbations in the ligand sphere. Both NEVPT2 and CASPT2 yield comparable mean absolute errors (MAEs) (429 cm^–1^ to 433 cm^–1^) once the systematic blue shift is corrected. This confirms that both schemes effectively recover the relative spacing of the 5f^2^ manifold. Nevertheless, NEVPT2 was adopted as the computational method for our subsequent analysis, since it offers a parameter-free formulation that avoids the need for empirical corrections, like the IPEA shift, which is often required in CASPT2 to prevent intruder state problems. NEVPT2 is also computationally more efficient with the large cc-pVQZ-X2C basis set, allowing the extension of the methodology to the sampled geometries. When the methodology was extended to the GMM-NEA convoluted spectra, the NEVPT2 scaling factor was slightly refined to 0.863 to match the experimental Band F position. Reproducing the position of Band F in the aqua ion ensures that the relative red-shifts observed upon chloride substitution are consistently captured.

The methodology relies on both configurational sampling and electronic structure methods. This is illustrated in the comparison of Wigner sampling and MD trajectories for the [PuO_2_(H_2_O)_5_]^2+^ aqua ion (Figure S7 of the SI), where the MD-derived spectrum is substantially broader than the sharp experimental band F that the Wigner approach reproduces. The observed discrepancies demonstrate the sensitivity of our spectroscopic model to differences in structural distribution.

The origin of the observed spectral differences is a direct consequence of the distance distributions shown in Figure S8 of the SI. MD trajectories sample a wider range of Pu–O equatorial distances, including a “tail” of distorted configurations that do not appear in the Wigner distribution. This arises from the potential energy surface (PES) used to parametrize the MD force field. Since this PES was constructed using the B3LYP functional, it struggles to fully describe the complex electron correlation effects inherent to the plutonyl center, which has been demonstrated to be crucial for a proper description of the structure and dynamics of actinides in solution.^26,86,96^ The treatment of water molecules is fundamentally different in both methods; whereas Wigner sampling captures the quantum vibrational ground state from the MP2 method, the MD simulation employs a rigid water model. The combination of these structural differences accounts for the enhanced bandwidth and the band displacement observed for Band F. The UV–vis spectrum can thus distinguish between these two sampling methods. These results confirm that Wigner sampling provides a reliable baseline for this system and justify its use to track the subtle spectral evolution with chloride complexation.

Given the agreement obtained with the NEVPT2 protocol for the aqua ion, we extended this scheme to the study of the [PuO_2_(H_2_O)_*m*_Cl_*n*_]^2–*n*^ series. Our goal is to evaluate the ability of the methodology to reproduce the systematic red-shift observed experimentally as the chloride concentration increases. To characterize the speciation of PuO_2_^2+^ in chloride media, the evolution of the experimental spectra was reconstructed by integrating multiple sources from the literature. The reference spectrum for the aqua ion was obtained by digitizing high-resolution data from Edelstein,^95^ particularly in the lower-intensity spectral regions. This digitized baseline was combined with a Gaussian function centered at the main absorption band, calibrated to a molar absorptivity of 550 M^–1^ cm^–1^ as established by Cohen^97^ and Gaunt et al.^32^ (see Figure S6 in the SI for the complete representation). The experimental absorption spectra for the aqua–chloro complexes were derived from the data reported by Runde et al.^6^ To account for the absence of absolute intensity units in the original data set, the molar absorptivity of the 1.5 M Cl^–^ spectrum was set to 145 M^–1^ cm^–1^, following the experimental values established by Lee et al.^98^ for the complex species at 1 M HCl. Adopting this specific intensity as a reference while strictly maintaining the internal relative proportions of the Runde et al.^6^ series, a consistent molar absorptivity scale was established for all chloride concentrations. This approach ensures that the systematic spectral evolution observed as a function of the ligand concentration remains comparable across different studies.

In Figure 7, the spectral evolution of Band F shows that increasing chloride concentration does not result in a simple shift of a single peak. Instead, the spectrum undergoes a dramatic transformation characterized by the sequential replacement of distinct bands. The narrow, dominant band of the aqua ion at 12,045 cm^–1^ (0 M Cl^–^) decreases in intensity, while new features grow in at lower energies. For intermediate chloride concentrations (1.5 to 5 M), a complex set of peaks is observed in the range of 11,800 cm^–1^ to 12,045 cm^–1^. At higher concentrations (7 to 16 M), these intermediate features merge and shift further, consolidating into a single, broader band envelope with its maximum at approximately 11,800 cm^–1^ at the highest salinity condition. These complex spectral changes, arising from highly sensitive f–f transitions, reflect the specific perturbation of the PuO_2_^2+^ equatorial ligand field as water molecules are progressively replaced by chloride ions. The fact that individual experimental spectra for pure intermediate species are unavailable, as they exist solely in equilibrium in solution, prevents a direct comparison with computed spectra of isolated complexes.

**Figure 7:**
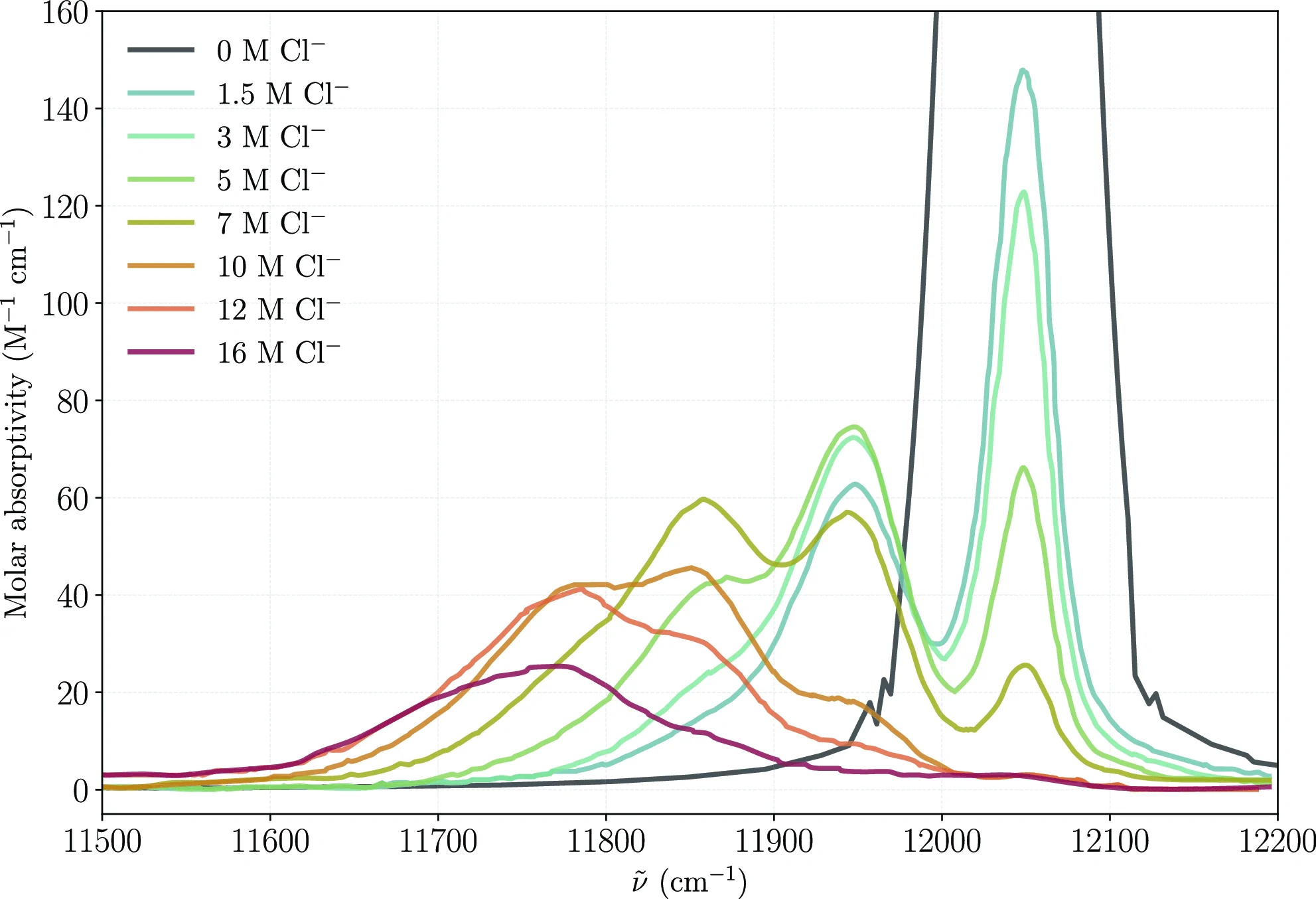
Evolution of the experimental spectra as a function of chloride concentration. The aqua ion spectrum (0 M Cl^–^, black line) was adapted from Edelstein,^95^ Copyright 2025 American Chemical Society, while the remaining spectra were adapted with permission from the work of Runde et al.,^22^ Copyright 1999 Elsevier.

Comparing the experimental series with the theoretical predictions in Figure 8 reveals a correlation with spectral shifts and specific coordination environments. For the cases of the dichloro and trichloro derivatives, two different isomers are possible. The calculated spectra represent the intermediate $\left[{PuO}_{2}{\left(H_{2}O\right)}_{3}{Cl}_{2}\right]$ and ${\left[{PuO}_{2}{\left(H_{2}O\right)}_{2}{Cl}_{3}\right]}^{-}$ species as statistically weighted averages of their isomers (using 85/15 and 95/5 ratios, respectively, shown in Figure S9), based on the Boltzmann distribution derived from their relative energies. The NEVPT2 calculations reproduce the experimental trend, with a systematic red-shift as each water molecule is substituted by chloride ligands in the equatorial plane. The main band for the aqua ion centered at 12,050 cm^–1^ shifts to 12,000 cm^–1^ for the monochloro derivative, to 11,900 cm^–1^ for the averaged dichloro complex, to 11,850 cm^–1^ for the averaged trichloro complex, and to 11,600 cm^–1^ for the tetrachloro derivative, but the absorptivity is ∼3 M^–1^ cm^–1^, almost 100 times smaller than the other derivatives.

**Figure 8:**
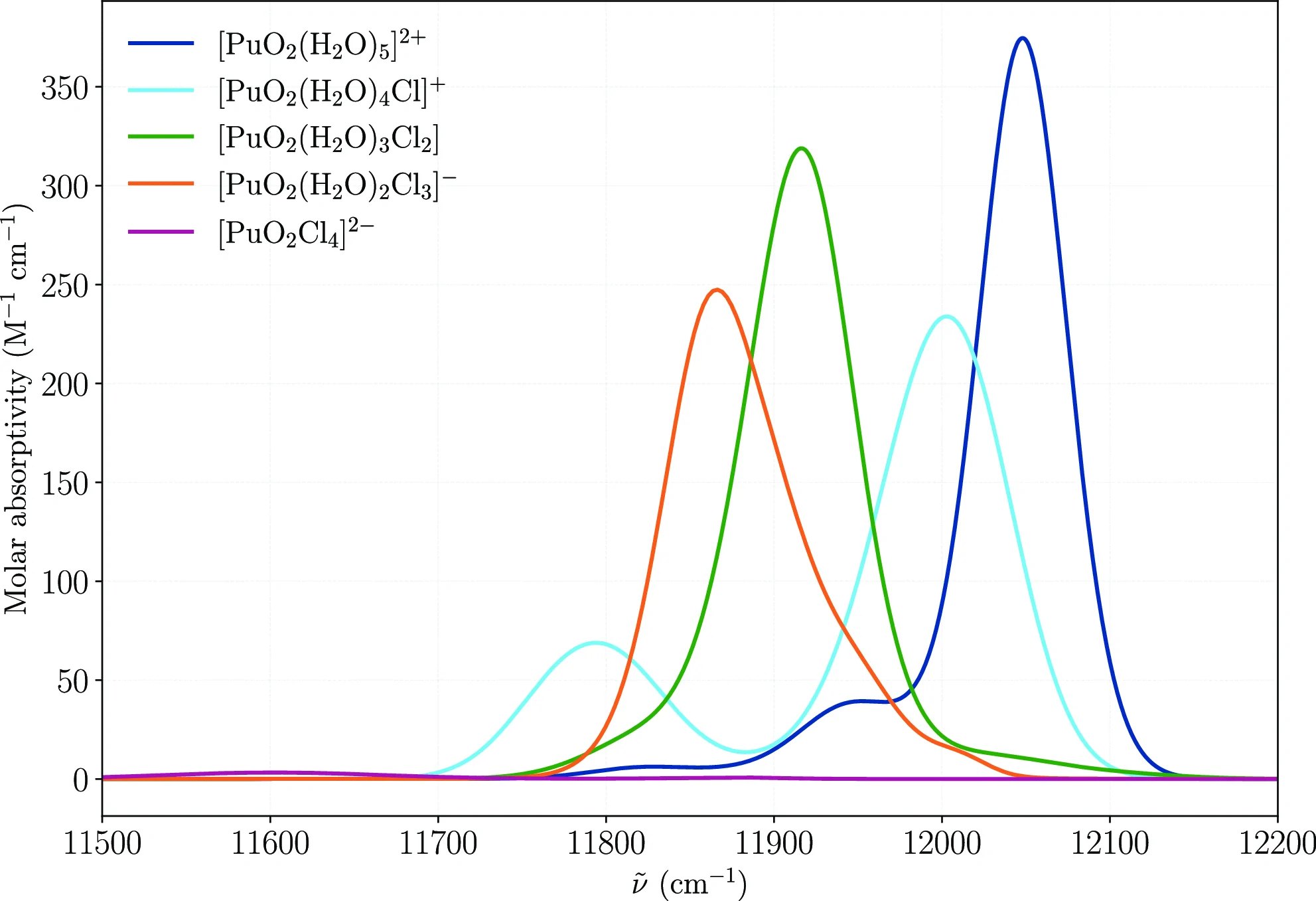
Evolution of the theoretical spectra with the increase of chloride anions in the first coordination shell of the PuO_2_^2+^ cation. The spectra of $\left[{PuO}_{2}{\left(H_{2}O\right)}_{3}{Cl}_{2}\right]$ and ${\left[{PuO}_{2}{\left(H_{2}O\right)}_{2}{Cl}_{3}\right]}^{-}$ are the result of weighted averages based on the relative populations of their isomers, 85/15 and 95/5, respectively.

Despite this qualitative agreement, some quantitative discrepancies persist. The simulated molar absorptivity of the aqua ion is approximately 30% lower than the experimental value (550 M^–1^ cm^–1^). The computed bands are also broader than the experimental ones, an effect likely coming from the sampling procedure and the Gaussian Mixture Model (GMM) methodology, although the GMM approach is intended to minimize the arbitrary assignment of half-widths. The theoretical bands are more closely spaced than those observed in the experimental evolution, suggesting that the current model underestimates relative energy differences between successive species.

The calculated intensity of the tetrachloride complex, [PuO_2_Cl_4_]^2–^, is negligible compared to the lower-order species. This reduced oscillator strength suggests a potential quenching of the transition probabilities, possibly due to the increased symmetry of the tetrachloride coordination environment or an insufficient description of the transition dipoles at this level of theory. This low computed intensity complicates a direct assignment of the experimental features at 16 M Cl^–^ to the tetrachloride species. However, at such extreme salinity, the presence of water in the first coordination shell is chemically improbable, and existing Raman experimental evidence is consistent with the formation of the tetrachloride complex. The theoretical methodology accurately tracks the energetic evolution across the full series, even though the precise spectral characterization of the highest-order complexes remains limited by the current model.

## Conclusions

In the [PuO_2_(H_2_O)_*m*_Cl_*n*_]^2–*n*^ series, the plutonyl core is highly sensitive to the equatorial ligand field. Progressive replacement of water by chloride elongates the Pu–O_yl_ bond through increased equatorial electron donation from the anionic ligands. The most pronounced structural reorganization is the transition from the pentacoordinated equatorial plane (D_5h_) to the tetracoordinated geometry (D_4h_) in the [PuO_2_Cl_4_]^2–^ complex, which relieves steric repulsion and provides a molecular basis for the vibrational signatures observed for the Raman B_2g_ mode at high salinity.

The MP2-Wigner sampled geometries reproduce the L_III_-edge EXAFS and XANES spectra with high fidelity. In the EXAFS spectra, the interference pattern between Pu–O_yl_ and equatorial Pu–OH_2_ or Pu–Cl paths in the *k* region between 7 Å^–1^ and 8 Å^–1^, constitutes a key structural fingerprint for chloride coordination. Consistent with the experimental features, XANES calculations confirm that the *trans*-dioxo geometry and Pu­(VI) oxidation state are preserved even when the equatorial shell undergoes significant electronic perturbation.

For the 5f^2^ electronic manifold, both NEVPT2 and CASPT2 effectively recover the relative energy spacing of the excited states from the [PuO_2_(H_2_O)_5_]^2+^-optimized structure. Both methods exhibit a systematic blue shift, partially corrected with the use of a scaling factor. NEVPT2 was adopted for its parameter-free formulation and the higher computational efficiency with the cc-pVQZ-X2C basis, enabling its application to the full Wigner ensemble.

The UV–vis spectrum shows high sensitivity to configurational sampling. Classical MD trajectories based on B3LYP parametrized force fields introduced excessive inhomogeneous broadening, while Wigner sampling successfully reproduced the sharp feature of the experimental band F. The difference between the two approaches makes the UV–vis spectrum a direct diagnostic of the quality of the structural ensemble.

The methodology successfully captures the experimental red-shift trend across the full substitution series, from 12,045 cm^–1^ in the aqua ion to the lower energy bands of the chloro complexes. The transition intensities for [PuO_2_Cl_4_]^2–^ are underestimated, resulting in a band at approximately 11,600 cm^–1^ of almost negligible intensity. Preliminary analysis shows that the intensity of the transition is stronger for the structures with more distorted geometries of the tetrachloro complex. However, the average of the samples cancels out these contributions. This means that adding anharmonic corrections to the Wigner sampling could help improve the structural fluctuations of plutonyl complexes, especially those with high symmetry. The computed energetic shifts allow for the individual contributions of each complex to be isolated, even for species that coexist in equilibrium without isolated experimental references.

## Supplementary Material



## Data Availability

The data set supporting the findings of this work is available in 10.12795/11441/186210.

## Notes

ORCA and FEFF input files; structural models for the aqua ion and the aqua–chloro complexes; comparison of experimental and theoretical EXAFS spectra for [PuO_2_(H_2_O)_5_]^2+^; EXAFS SS contributions for [PuO_2_(H_2_O)_5_]^2+^; experimental^21^ Pu M4-edge XANES spectrum of PuO_2_^2+^ in aqueous solution and 6 M hydrochloric acid; theoretical Pu M4-edge XANES spectrum of [PuO_2_(H_2_O)_5_]^2+^ and ${\left[{PuO}_{2}{\left(H_{2}O\right)}_{2}{Cl}_{3}\right]}^{-}$ ; evolution of experimental UV–vis spectra shown; comparison of UV–vis for [PuO_2_(H_2_O)_5_]^2+^ obtained from MD and Wigner sampling; Pu–O and O–H distance distribution; and contribution of isomers of ${\left[{PuO}_{2}{\left(H_{2}O\right)}_{3}{Cl}_{2}\right]}^{2+}$ and ${\left[{PuO}_{2}{\left(H_{2}O\right)}_{2}{Cl}_{3}\right]}^{-}$ to the UV–vis (PDF)
