# Supplementary material for: Aqua–Chloro Complexes of Plutonyl in Hydrochloric Aqueous Solutions: Theoretical Study of Individual Contributions to XAS and UV–Vis Spectra
Source: Inorg Chem. 2026 Aug 9;65(33):19417–28. doi: 10.1021/acs.inorgchem.6c02837 (PMC13509013; doi:10.1021/acs.inorgchem.6c02837)
Supplement: Supplementary file 1 [file ic6c02837_si_001.pdf]

Supporting Information: Aqua-chloro complexes of plutonyl in hydrochloric aqueous solutions: theoretical study of individual contributions to XAS and UV-Vis spectra

Gema Raposo-Hernández,† Rafael R. Pappalardo,† Andrea Melchior,‡ and Enrique Sánchez Marcos*,†

†*Department of Physical Chemistry, University of Seville, 41012-Seville, Spain*

‡*Dipartimento Politecnico di Ingegneria e Architettura, Laboratorio di Tecnologie Chimiche, Università di Udine, 33100-Udine, Italy*

E-mail: sanchez@us.es

ORCA input

```
! x2c-TZVPall RIJK
! NEVPT2 X2C
! MOREAD
%moinp "x0001_5w_cpcm_7f.gbw"
%maxcore  4000
%pal nprocs 12 end
%method
  FrozenCore -92
End
%casscf
 nel 2
 norb 7
 mult 3,1
 nroots 21,28
 rel
   dosoc true #spin-orbit coupling (and ZFS)
 end
end

%scf
guessmode = cmatrix
end

%basis
NewAuxGTO Pu  "autoaux" end
NewAuxGTO O  "autoaux" end
NewAuxGTO H  "autoaux" end
NewAuxJKGTO O  "autoaux" end
NewAuxJKGTO H  "autoaux" end
NewAuxJKGTO Pu "autoaux" end
#!----------------------------------------------------------------------
#! Basis Set Exchange
#! Version 0.12
#! https://www.basissetexchange.org
#!----------------------------------------------------------------------
#!   Basis set: cc-pVQZ-X2C
#! Description: cc-pVQZ-X2C
#!        Role: orbital
#!     Version: 1  (Data from ccRepo)
#!----------------------------------------------------------------------
NewGTO Pu
*Pu basis set*
end
```

```
end
* xyz 2 3
Pu -0.001654 0.014237 0.025416
O 1.771254 0.027041 -0.045079
O -1.736386 -0.033434 0.010019
O -0.093798 2.365158 -0.275887
H 0.747812 2.835601 -0.237359
H -0.492777 2.981830 0.480422
O 0.004896 0.852883 2.088714
H 0.423761 1.649879 2.628419
H -0.896639 0.887116 2.759984
O 0.102868 -1.852349 1.549837
H 0.847716 -2.245253 1.886017
H -0.551778 -2.701401 1.720604
O 0.065303 -2.071696 -1.334284
H 0.265254 -2.925864 -0.819354
H -0.628806 -2.369607 -2.154037
O -0.047699 0.550801 -2.381387
H 0.417248 0.051826 -3.424814
H -0.785649 0.953228 -2.834660
*
```

EXAFS input

```
TITLE_Pu
EDGE L3
*        mphase,  mpath,  mfeff,  mchi
CONTROL  1   1   1   1   1   1
PRINT      0       0       0       1

EXCHANGE 0 -2.0 0
SCF 6.0

COREHOLE RPA
TDLDA  1
CRITERIA 4.0  2.5

RPATH   6.0  ! ONLY for EXAFS
NLEG    4

S02 1.0

POTENTIALS
       0    94   Pu    3   3
       1    8    Oyl   3   3
       2    8    O     3   3
       3    1    H     2   2
ATOMS
   0.0040150    0.0158470    0.0131100  0   Pu     0.0209552  0
   1.6831130   -0.0746830    0.0390230  1    O     1.6852210  0
  -1.7305730   -0.1432140    0.0744030  1    O     1.7380820  0
  -0.0645440    2.5174100   -0.3261470  2    O     2.5392698  0
   0.7065100    3.1403020   -0.4434600  3    H     3.2492014  0
  -0.6951670    2.6935600    0.6034560  3    H     2.8465210  0
  -0.0485240    0.9386360    2.1863080  2    O     2.3797762  0
   0.7114950    1.1836080    2.5189890  3    H     2.8727093  0
  -0.5019780    0.6217510    2.9630410  3    H     3.0689034  0
   0.1179140   -1.8737670    1.4554670  2    O     2.3755611  0
   0.8167220   -1.6737550    2.1234320  3    H     2.8244387  0
  -0.7182230   -2.3156510    1.8541360  3    H     3.0521966  0
   0.0677430   -2.1022060   -1.3467430  2    O     2.4975139  0
   0.1788570   -3.1159540   -0.9019310  3    H     3.2487903  0
  -0.5505990   -2.1817870   -2.0240240  3    H     3.0265536  0
  -0.0774060    0.5149550   -2.3172060  2    O     2.3749977  0
   0.4629350    0.2681090   -3.2138770  3    H     3.2580971  0
  -0.5531890    1.0799120   -2.9253960  3    H     3.1670443  0
END
```

XANES input

```
TITLE  PuO22+
HOLE 4   1.0     Pu  L3-edge

XANES 6 0.10 0.
FMS 6.0 1
OPCONS
MPSE 2
COREHOLE RPA
*TDLDA 1
SCF 6.0 1
EXCHANGE  0 0. 0.03
RGRID 0.01
EGRID
e_grid -60 150 2

 POTENTIALS
 *   ipot   z  label  l
       0    94  Pu    3   3
       1    8   O     3   3
       2    8   O     3   3
       3    1   H     2   2

*         mphase,  mpath,  mfeff,  mchi
CONTROL    1       1       1       1       1      1
PRINT      0       0       0       3

*S02 1.0
ATOMS
   0.0040150    0.0158470    0.0131100  0   Pu     0.0209552  0
   1.6831130   -0.0746830    0.0390230  1    O     1.6852210  0
  -1.7305730   -0.1432140    0.0744030  1    O     1.7380820  0
  -0.0645440    2.5174100   -0.3261470  2    O     2.5392698  0
   0.7065100    3.1403020   -0.4434600  3    H     3.2492014  0
  -0.6951670    2.6935600    0.6034560  3    H     2.8465210  0
  -0.0485240    0.9386360    2.1863080  2    O     2.3797762  0
   0.7114950    1.1836080    2.5189890  3    H     2.8727093  0
  -0.5019780    0.6217510    2.9630410  3    H     3.0689034  0
   0.1179140   -1.8737670    1.4554670  2    O     2.3755611  0
   0.8167220   -1.6737550    2.1234320  3    H     2.8244387  0
  -0.7182230   -2.3156510    1.8541360  3    H     3.0521966  0
   0.0677430   -2.1022060   -1.3467430  2    O     2.4975139  0
   0.1788570   -3.1159540   -0.9019310  3    H     3.2487903  0
  -0.5505990   -2.1817870   -2.0240240  3    H     3.0265536  0
```

```
  -0.0774060    0.5149550   -2.3172060  2    O     2.3749977  0
   0.4629350    0.2681090   -3.2138770  3    H     3.2580971  0
  -0.5531890    1.0799120   -2.9253960  3    H     3.1670443  0
END
```

Structural results

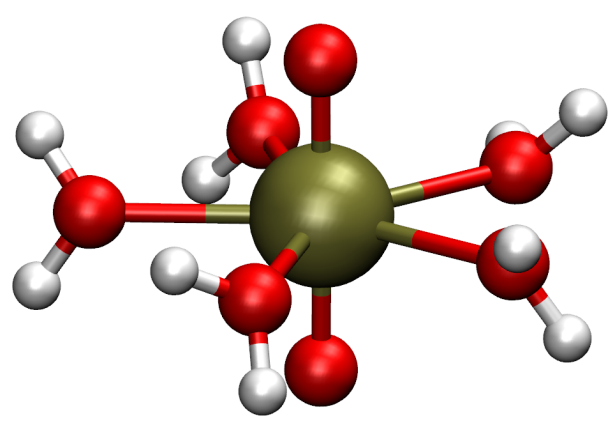

(a) $[PuO_2(H_2O)_5]^{2+}$

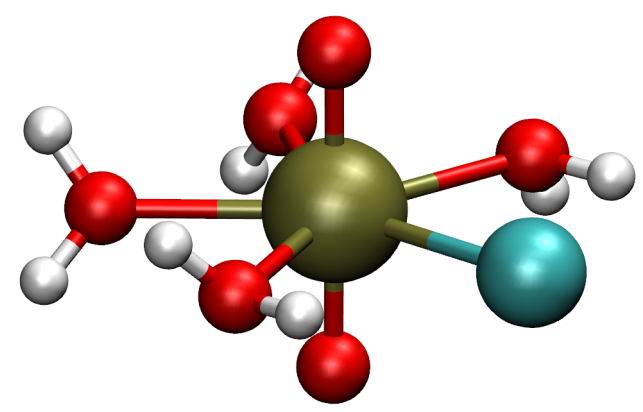

(b) $[PuO_2(H_2O)_4Cl]^{+}$

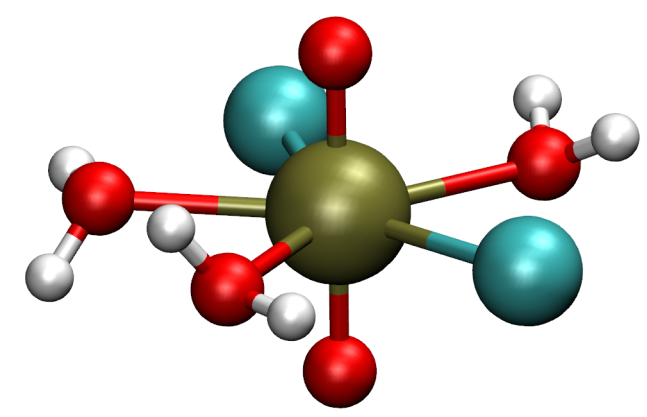

(c) $[PuO_2(H_2O)_3Cl_2]$ (trans)

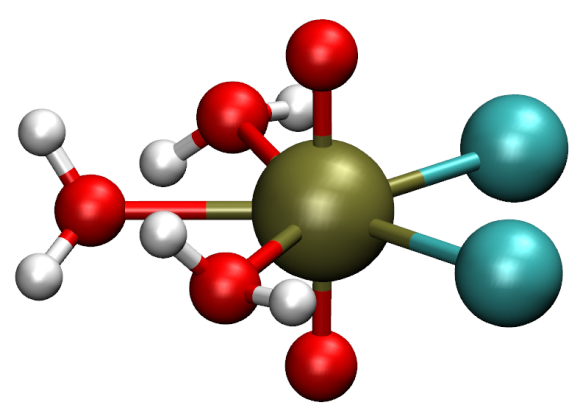

(d) $[PuO_2(H_2O)_3Cl_2]$ (cis)

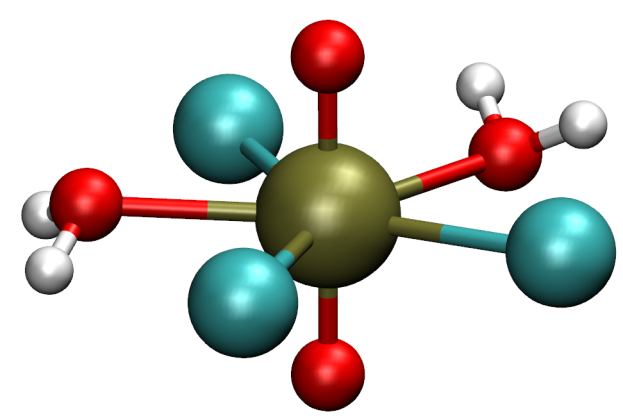

(e) $[PuO_2(H_2O)_2Cl_3]^{-}$ (trans)

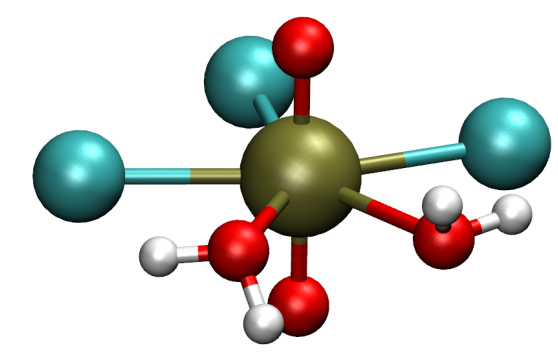

(f) $[PuO_2(H_2O)_2Cl_3]^{-}$ (cis)

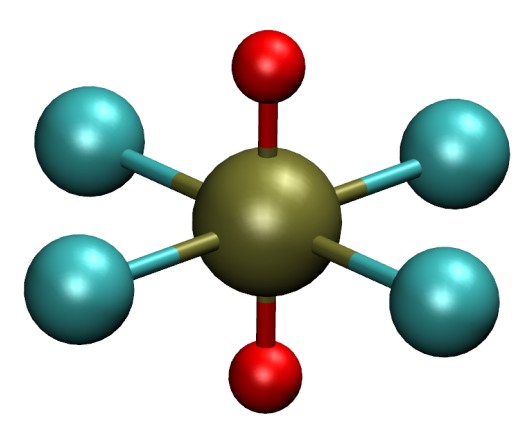

(g) $[PuO_2Cl_4]^{2-}$

Figure S1: Optimized geometries of the $[PuO_2(H_2O)_mCl_n]^{2-n}$ series.

EXAFS results

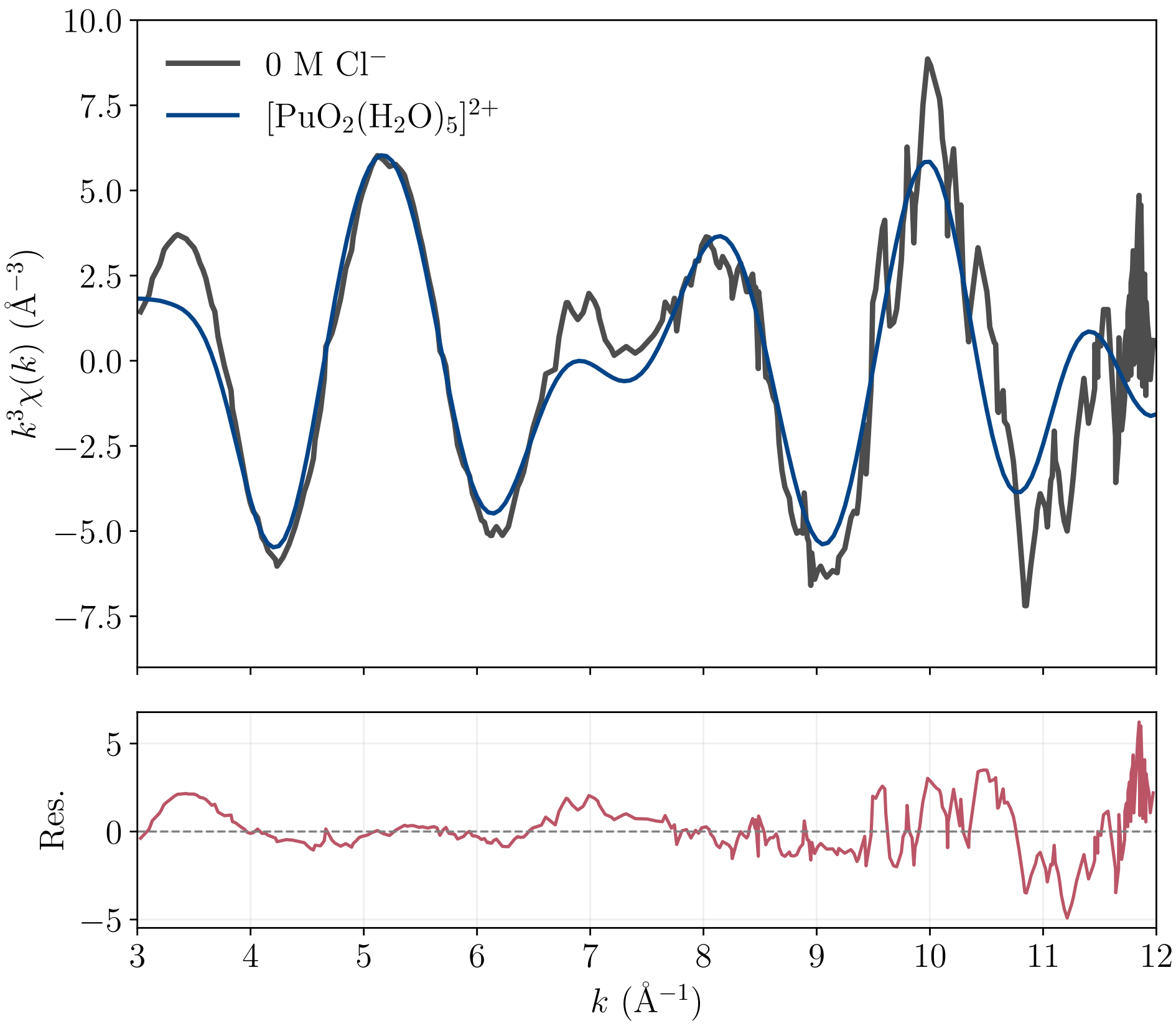

Figure S2: Experimental[S1] (black) and theoretical (dark blue) EXAFS spectrum of PuO_2^{2+} in solution. At the bottom, in red, the residuals.

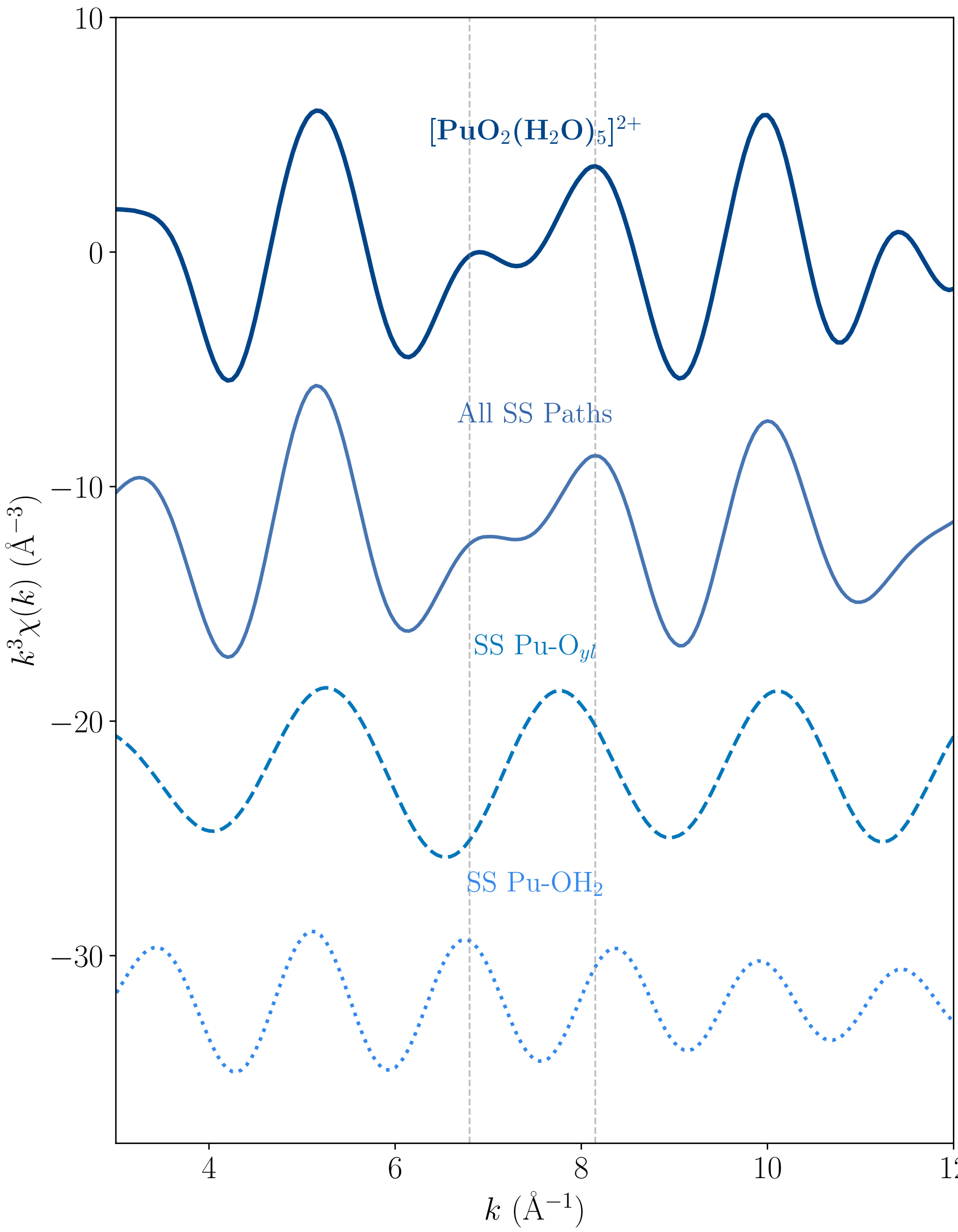

Figure S3: Theoretical EXAFS spectrum of $[PuO_2(H_2O)_5]^{2+}$ and the contribution of single scattering paths.

XANES results

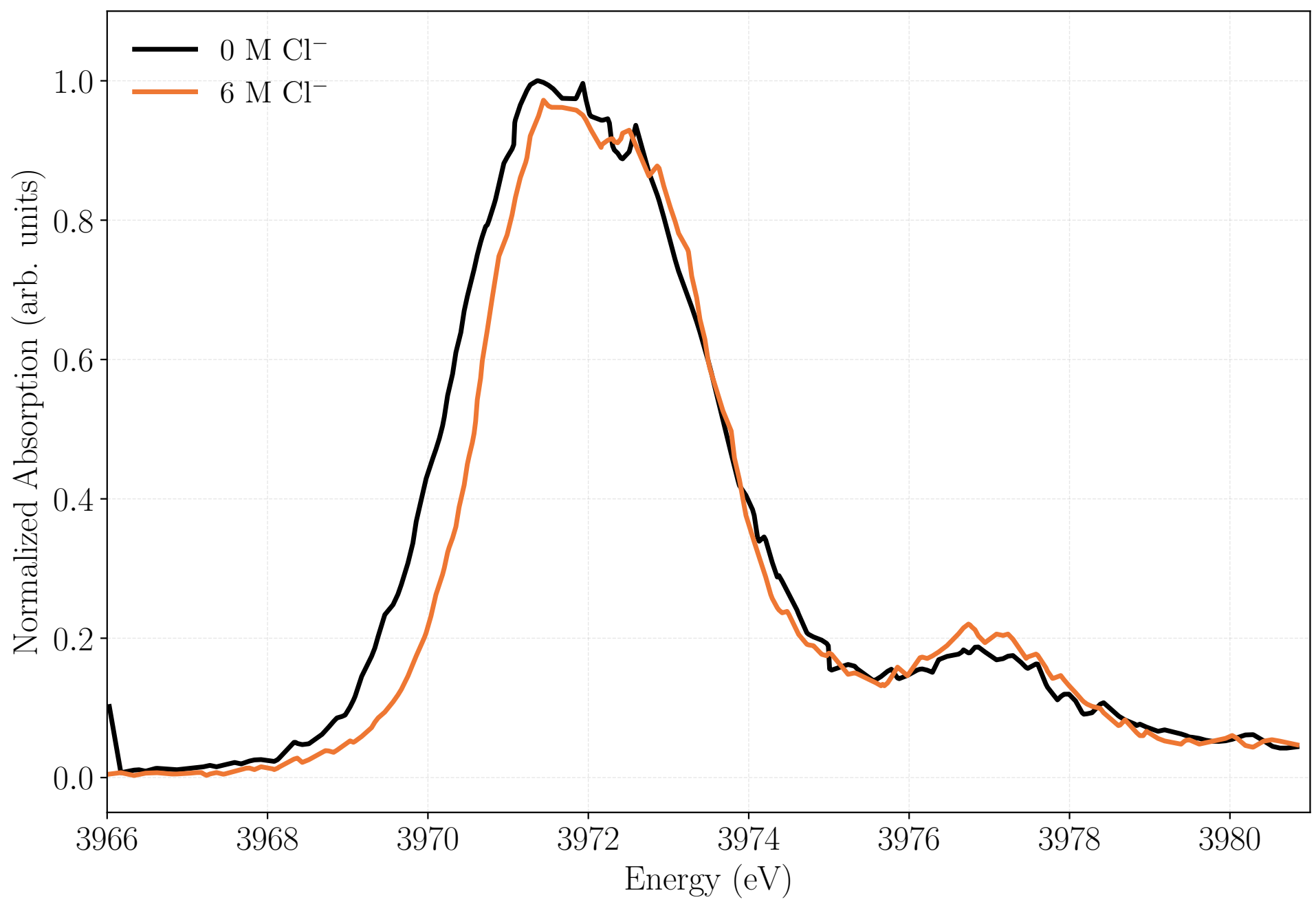

Figure S4: Experimental[S1] Pu M4-edge XANES spectrum of PuO_2^{2+} in aqueous solution and 6M hydrochloric acid.

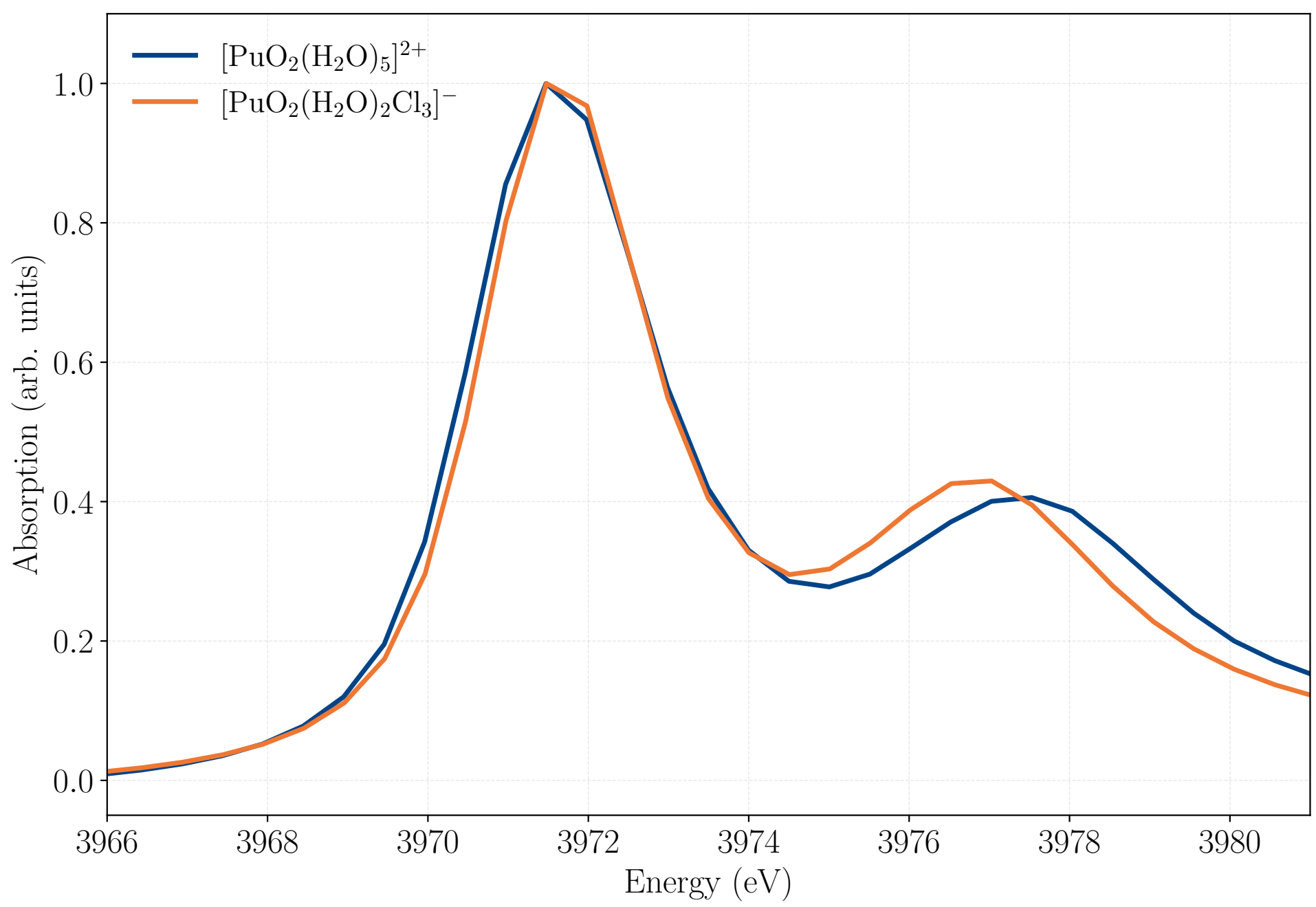

Figure S5: Theoretical Pu M4-edge XANES spectrum of $[PuO_2(H_2O)_5]^{2+}$ and $[PuO_2(H_2O)_2Cl_3]^{-}$

UV-Vis results

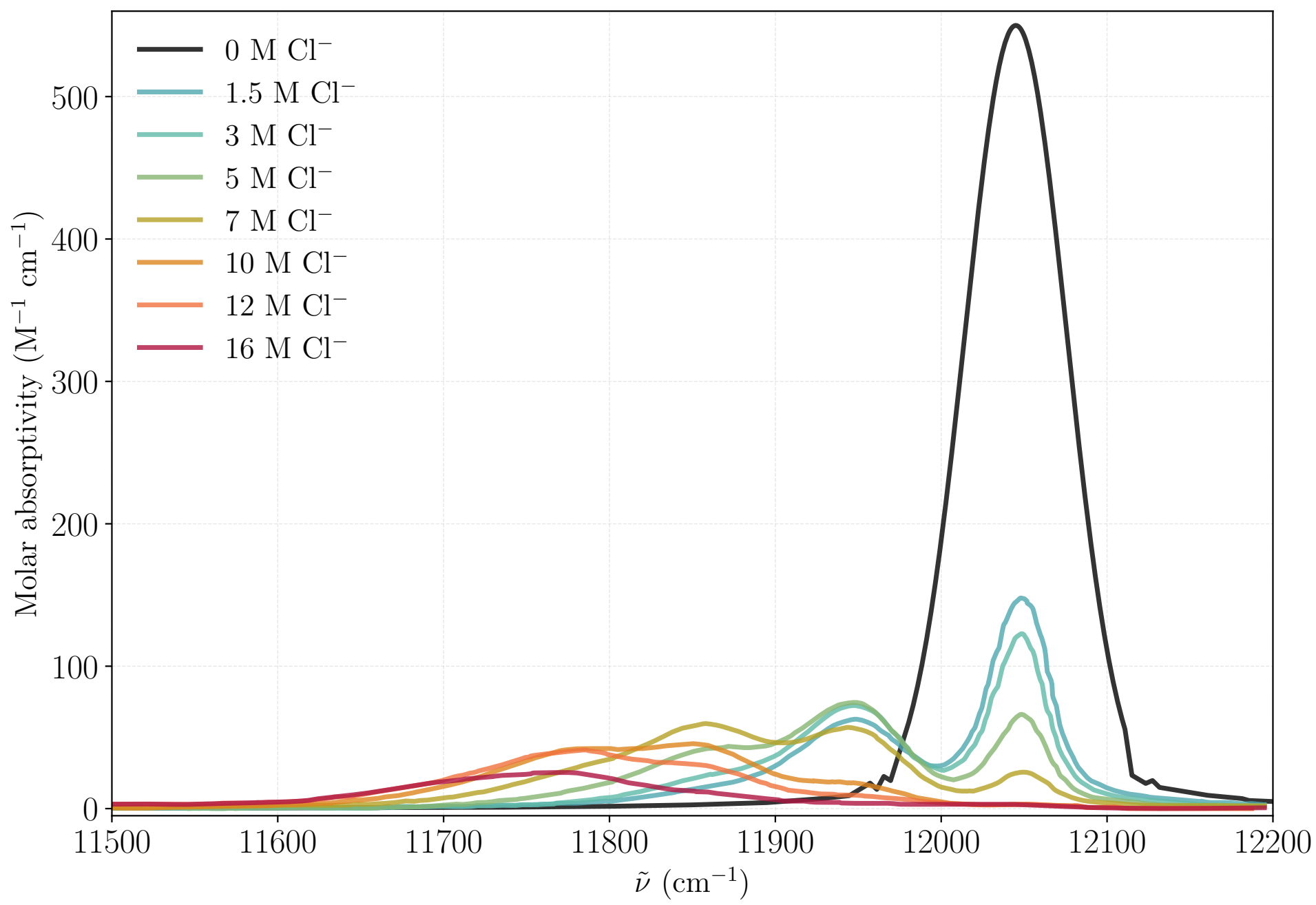

Figure S6: Evolution of the experimental spectra as a function of chloride concentration. The aqua ion spectrum (0 M Cl^-, black line) was obtained from Edelstein[S2], while the remaining chlorinated spectra were digitalized from the work of Runde *et al.*[S3].

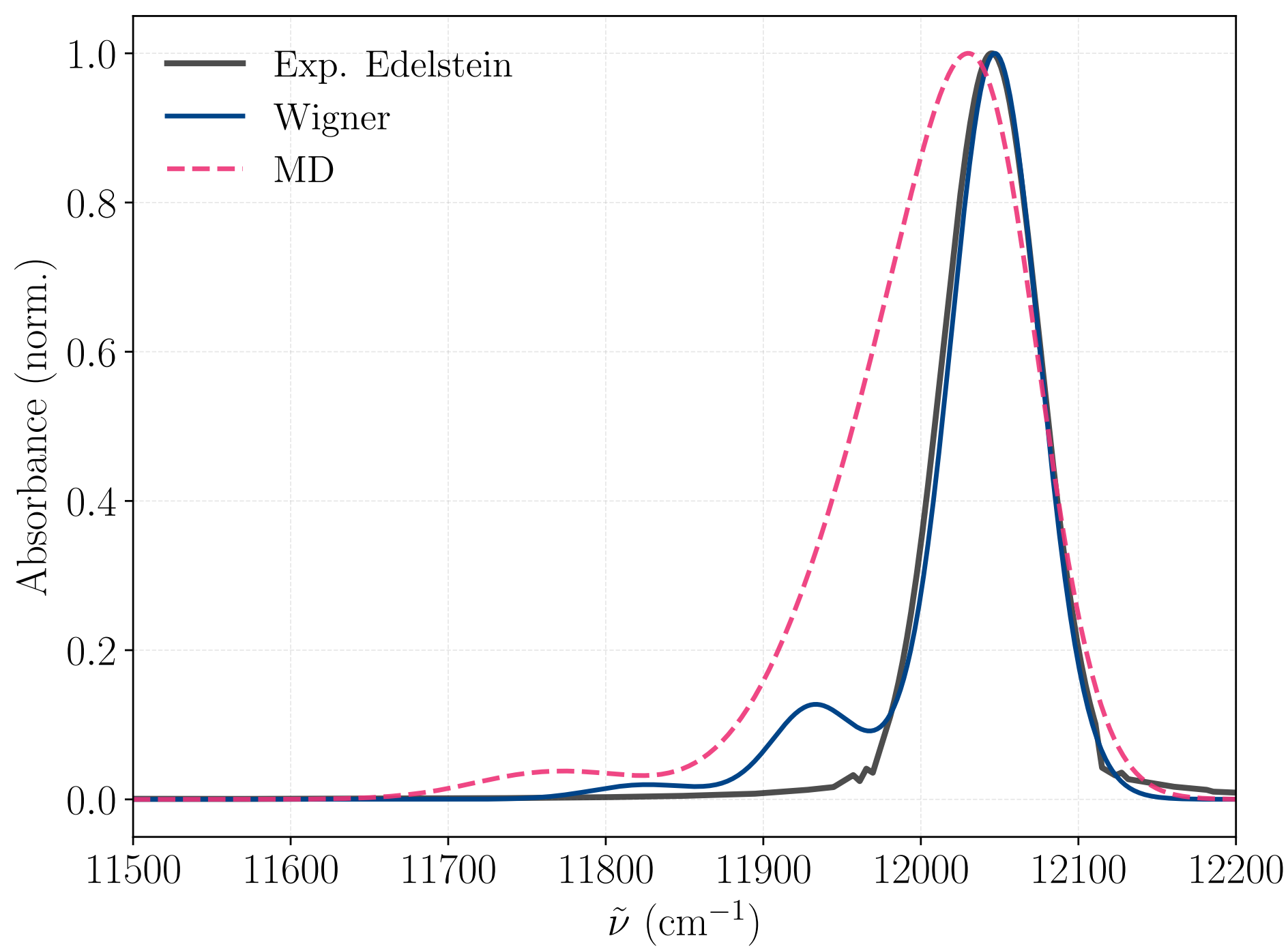

Figure S7: Comparison of the normalized computed spectra for Wigner (blue) and MD (pink) sampling methods using the same scaling factors and level of theory. It is compared with the spectrum extracted from Edelstein[S2] (black).

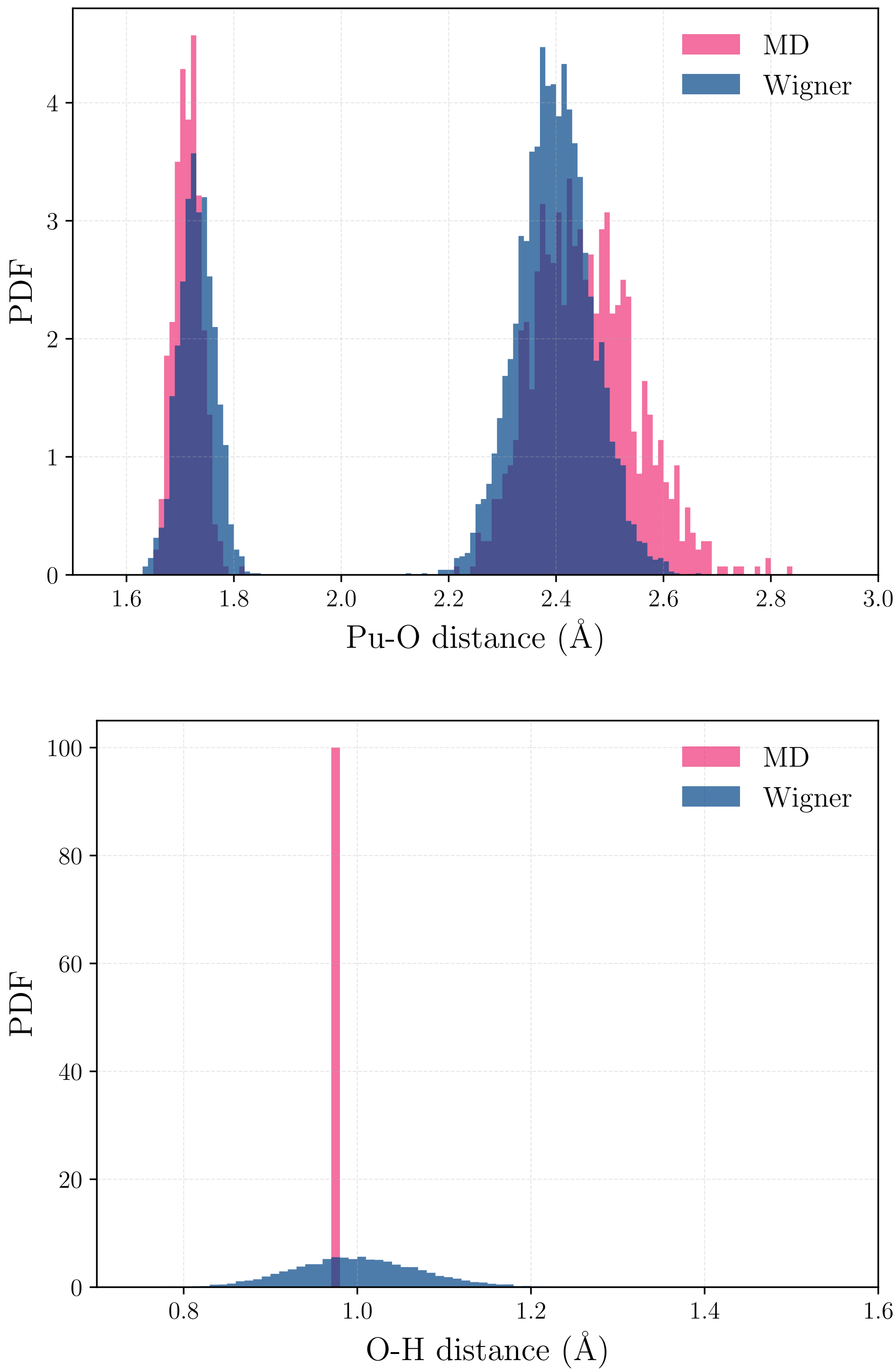

Figure S8: Distribution of Pu−O and O−H distances (Probability Density Function, PDF) for the $[PuO_2(H_2O)_5]^{2+}$. Comparison between MD simulation and Wigner distribution.

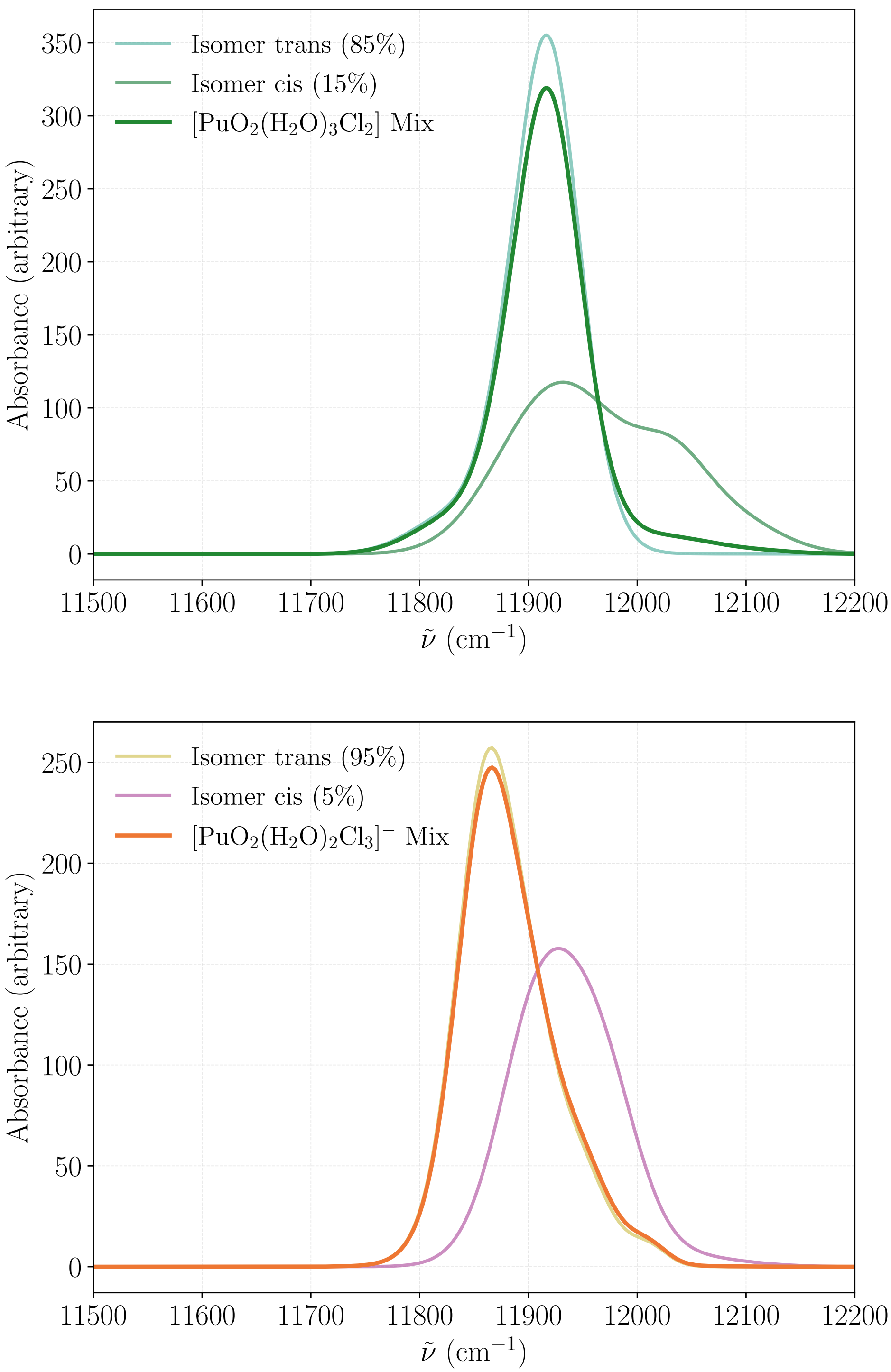

Figure S9: Mixture of the two possible isomers based on relative energies of the optimized structures.

References

(S1) Neill, T. S.; Ramanantoanina, H.; Palina, N.; Prüßmann, T.; Rothe, J.; Gaona, X.; Fellhauer, D.; Schild, D.; Wansorra, C.; Hauschild, D.; Steininger, R.; Schacherl, B.; Heske, C.; Weinhardt, L.; Walter, O.; Weigend, F.; Vitova, T. The Role of Halides in the Bonding and Electronic Structure of Actinyl(VI) Halides-Energy Match Driven Stability. *J. Am. Chem. Soc.* **2025**, *147*, 35401–35412, DOI: 10.1021/jacs.5c08999.

(S2) Edelstein, N. M. Revisiting the Optical Spectrum of the Plutonyl Ion $(PuO_2)^{2+}$ in 1 M $HClO_4$. *J. Phys. Chem. A* **2025**, *129*, 50–57, DOI: 10.1021/acs.jpca.4c05837.

(S3) Runde, W.; Neu, M. P.; Conradson, S. D.; Clark, D. L.; Palmer, P. D.; Reilly, S. D.; Scott, B. L.; Tait, C. D. Spectroscopic Investigation of Actinide Speciation in Concentrated Chloride Solution. *MRS Proc.* **1996**, *465*, 693, DOI: 10.1557/PROC-465-693.
